# When algorithmic managers fail to fulfill their promises: The role of anthropomorphism in shaping justice perceptions

**DOI:** 10.1371/journal.pone.0340860

**Published:** 2026-02-20

**Authors:** Kyriaki Fousiani, Lorenz Reinold Cremer, Mehmet Çetin, Sabine Raeder

**Affiliations:** 1 Department of Psychology, University of Groningen, Groningen, The Netherlands; 2 Department of Leadership and Organization, Kristiania University College, Oslo, Norway; 3 Department of Psychology, University of Oslo, Norway; Montclair State University, UNITED STATES OF AMERICA

## Abstract

This study explored how employees perceive distributive justice (i.e., perceived fairness of an outcome) when algorithmic managers fail to fulfill their promises. Moreover, drawing on anthropomorphism theory, we further investigated the moderating role of the anthropomorphism of algorithmic managers. We hypothesized that employees perceive lower distributive justice when their algorithmic manager fails to fulfill transactional rather than relational promises, especially when the manager is not anthropomorphized. We conducted two vignette experiments to test our hypotheses. In both Study 1 (*N* = 258 employees; *M*age = 36.76) and Study 2 (*N* = 248 employees; *M*age = 37.12), a 2 (type of nonfulfilled promises: relational versus transactional) × 2 (algorithmic manager’s anthropomorphism: high versus low) between-subjects design was employed. Study 2 (preregistered) further examined the mediating role of the perceived rigidity of the algorithmic manager in the hypothesized relationships. Study 1 showed that employees perceive lower distributive justice when algorithmic managers fail to fulfill transactional (as opposed to relational) promises, especially when managers are not anthropomorphized. In Study 2, we found that under these conditions, algorithmic managers are perceived as more rigid, which in turn is related to lower perceived distributive justice. These findings highlight the benefits of adding human-like traits to algorithmic management systems to reduce negative reactions when such systems fail to fulfill their commitments. However, ethical concerns arise about encouraging employees to treat algorithmic managers as humans, as anthropomorphization may blur boundaries and undermine accountability.

## When algorithmic managers fail to fulfill their promises: The role of anthropomorphism in shaping justice perceptions

As technologies such as machine learning and robotics rapidly advance, algorithmic management is increasingly used across industries to allocate tasks, monitor performance, and make personnel decisions [1]. While algorithmic management, such as the use of artificial intelligence (AI) and algorithms to perform managerial functions that are traditionally handled by human managers [2], is praised for its efficiency and objectivity [3,4], it is often criticized for its rigidity and lack of human discretion, which can generate perceptions of unfairness and injustice [5–7]. Indeed, algorithmic systems’ data-driven decisions may lack contextual sensitivity, leading employees to view them as inflexible and unjust [8,9]. One central concern, therefore, is how fair employees perceive the outcomes they receive from algorithmic managers [10,11].

Justice concerns are especially salient in the face of negative outcomes, as individuals are generally more sensitive to unfairness when they experience unfavorable rather than positive events [12–14; see also “negativity bias”; 15]. In line with this “negativity bias,” our study focuses on distributive justice—employees’ perceptions of the fairness of outcomes—when algorithmic managers fail to meet expectations.

Traditionally, algorithmic management has been used for transactional functions such as scheduling or performing monitoring [16]. However, recent technological developments have led to the expansion of algorithmic management into more relational areas, such as mentoring, employee engagement, and emotional support [10,17]. Importantly, employees typically associate relational functions and promises (i.e., promises focusing on ongoing support and long-term benefits) with human managers, whereas transactional ones (i.e., promises focusing on specific, task-oriented exchanges and immediate rewards [18]) are seen as relevant to both human and algorithmic managers; thus, there is no inherent human bias regarding expectations for transactional promises [17]. However, little is known about how employees respond when algorithmic managers fail to fulfill these distinct types of promises. Because algorithmic systems lack human-like intent or discretion [19], failing to fulfill relational versus transactional promises may elicit different justice appraisals.

Moreover, algorithmic managers are often designed with human-like features to enhance trust and engagement. These features can be both form-related, such as names or avatars, and behavioral, such as conversational interfaces [20]. This anthropomorphism (i.e., the attribution of human-like qualities to nonhuman agents [21]) can shape how employees interpret fairness-related outcomes. While greater anthropomorphism may buffer negative reactions by making systems appear more intentional or understanding [22,23], it remains unclear whether these effects extend to justice perceptions when algorithmic managers fail to fulfill relational or transactional promises.

Although both anthropomorphism theory [21] and organizational justice theory [24] have been extensively studied, prior research has rarely examined how these frameworks jointly explain fairness perceptions in the context of algorithmic management. Existing studies have compared primarily human and algorithmic managers (e.g., [17,25]), leaving unclear how employees evaluate different types of psychological contract nonfulfillment (relational vs. transactional) by algorithmic managers themselves and how anthropomorphic design features shape these evaluations. Furthermore, while justice theories emphasize outcome fairness, little attention has been given to the appraisal mechanisms—such as perceived rigidity—that underlie fairness judgments toward nonhuman agents. Our study addresses these gaps by integrating insights from psychological contract, anthropomorphism, and justice literatures to examine how nonfulfillment of relational versus transactional promises by algorithmic managers affects employees’ distributive justice perceptions and how anthropomorphic cues modulate these effects. In doing so, we extend existing theories by specifying the expectation–appraisal–justice process in algorithmic management and by showing how anthropomorphism influences fairness appraisals in a novel, AI-driven managerial context.

This study makes three significant theoretical contributions. First, it extends research on employee responses to algorithmic management (e.g., [1,17]) by examining how the nonfulfillment of relational versus transactional promises by algorithmic managers shapes employees’ distributive justice perceptions. While prior work suggests that employees more readily associate relational promises with human managers [17], we test how failing to fulfill both promise types by algorithmic managers is evaluated, revealing potential biases in employees’ assumptions about algorithms’ capacity to manage relational promises. Because our focus is on the outcomes of unmet promises (rather than procedures or interpersonal treatment), our focal construct is distributive justice rather than procedural or interactional justice.

Second, this study applies anthropomorphism theory [21] to explore how human-like qualities in algorithmic management systems might mitigate the negative effects of algorithmic managers’ failure to fulfill promises. By comparing highly anthropomorphic algorithmic managers with less anthropomorphic ones, we offer new insights into how the human-like characteristics of algorithmic systems can influence employee perceptions of distributive justice and enhance the acceptance of algorithmic management. Third, this study contributes to the organizational justice literature [24] and its connection with AI [3,11] by highlighting the specific challenges that arise when employees experience unfulfilled promises by algorithmic managers [25]. Understanding how employees interpret negative outcomes (e.g., nonfulfillment of managers’ promises) in their interactions with algorithmic management can significantly influence their trust in these systems, their engagement with their work, and their overall satisfaction with organizational decision-making.

### Algorithmic management and perceived distributive justice

Employees often experience negative interactions with algorithmic managers, which can profoundly influence their perceptions of organizational justice [3]. In their review, Noponen et al. noted that algorithmic management systems, while potentially increasing efficiency and autonomy, often reinforce Tayloristic control (i.e., overemphasizing the efficiency and close supervision of workers, often at the expense of worker autonomy [26]), leading to perceptions of unfairness among employees [11]. Similarly, Zhang et al. noted how the rigidity of algorithmic management can heighten stress and reduce perceived autonomy, triggering deviant behaviors among gig workers who feel deprived of agency [27]. Lu et al. further supported this finding by demonstrating that the opaque nature of algorithmic management can negatively impact organizational commitment [7]. Finally, Birhane emphasized the related ethical implications, advocating for a relational and context-sensitive approach to algorithmic management to counteract inherent biases and ensure fairer outcomes for workers [28]. Together, these studies suggest that while algorithmic management can optimize efficiency, it is widely associated with the perception of unfairness. Importantly, however, employees’ negative responses to algorithmic management are not uniform or unconditional. Prior research shows that algorithms are often appreciated for their precision, consistency, and perceived objectivity [29], particularly when they deliver accurate or predictable outcomes. Thus, employees’ fairness judgments are likely to depend on whether algorithmic managers meet or fail these expectations of competence.

Relatedly, a well-established body of research has demonstrated that individuals are generally more sensitive to injustice when they experience negative rather than positive outcomes [12–14], which is irrespective of whether decisions are made by human or algorithmic agents. This asymmetry, often attributed to so-called “negativity bias” [15], suggests that individuals scrutinize decision outcomes more critically when they are unfavorable than when they are favorable. In the case of algorithmic management, however, the perceived rigidity and inflexibility of algorithms (see also [30]) may exacerbate justice concerns when outcomes are unfavorable (see [25], see also [31]), whereas no such bias against algorithmic management is observed when outcomes are positive.

In this study, we examine how employees respond in terms of justice perceptions when algorithmic managers fail to fulfill promised inducements, leading to unfavorable outcomes. When expectations regarding rewards or benefits are violated, employees evaluate whether they have received what they perceive as a fair share [12,32]. Accordingly, we focus on *distributive justice*—the perceived fairness of outcomes and resource distributions (e.g., pay, rewards [32,33])—as it is most directly relevant to employees’ reactions to the unfavorable outcomes of algorithmic decision-making.

While organizational justice research also identifies procedural justice (i.e., the perceived fairness of decision-making processes) and interactional justice (i.e., the perceived fairness of interpersonal treatment [12,32]), both of which are undoubtedly relevant in the context of algorithmic management, their evaluation typically requires direct engagement between employees and the decision-making entity to assess aspects such as transparency, voice, and respect [32,33]. In contrast, distributive justice judgments—concerned with the fairness of decision outcomes—can form immediately upon receiving an outcome, making them particularly salient when examining justice perceptions in response to unfavorable decisions made by algorithmic managers. Accordingly, our study focuses exclusively on distributive justice, specifically on employees’ fairness perceptions of negative outcomes under algorithmic management.

### The impact of algorithmic managers’ failure to fulfill relational versus transactional promises on perceived justice

According to the psychological contract literature, employees often perceive a set of implicit promises or commitments from their organization that extend beyond formal written contracts and that are typically communicated through their managers as organizational representatives [18]. These promises include secure employment, competitive salaries, career advancement opportunities, and support for personal issues [34]. The psychological contracts literature has identified two main types of promises that managers may fulfill or fail to fulfill: relational and transactional [18]. *Relational* promises focus on long-term, socioemotional exchanges that enhance a sense of care, recognition, and future investment. These promises help build an emotional connection, which can positively influence employees’ attitudes and behaviors toward the organization [17,35] (see also [36,37]). Conversely, *transactional* promises are shorter-term and focused on economic exchanges, such as monetary rewards, promotion schemas, or specific tasks. These are often linked to more formal, impersonal relationships and are typically seen in environments in which employees may experience weaker emotional ties with the employer [18,38]. Consistent with extensive meta-analytic evidence, psychological contract violation has been shown to strongly predict aversive employee attitudes and behaviors, including reduced job satisfaction, organizational commitment, and trust, as well as increased turnover intentions and deviant behavior (e.g., [39]). Crucially, when managers fail to fulfill these promises, whether they are relational or transactional, employees tend to experience lower distributive, procedural, and interactional justice [40].

In the context of algorithmic management, recent research has suggested that employees distinctly associate relational promises primarily with human managers, whereas transactional promises are expected equally from both human and algorithmic managers [17]. This distinction arises because relational promises often require contextual understanding, intent, moral judgment, and genuine interpersonal engagement, which are perceived as inherently human capabilities [41,42]. Interestingly, in roles for which inherently human capabilities are needed, employees are more likely to accept decisions and perceive them as fairer, more trustworthy and legitimate when they are made by human managers than when they are made by algorithmic systems [19,43]. In contrast, transactional promises are viewed as standardized and rule-based, making them more easily managed by algorithms (besides humans). The clear and structured nature of such tasks aligns well with the automated, data-driven processes of algorithmic management systems, which are perceived as efficient and objective in handling routine, transactional responsibilities [27]. Nevertheless, algorithmic management is increasingly being integrated into both transactional and relational responsibilities in real-world settings (e.g., [1,17,44,45]). This growing reliance on AI-driven management for transactional and relational responsibilities underscores the importance of understanding how employees perceive justice when promises are not fulfilled [46].

Considering the distinct nature of transactional and relational promises and the expectation that algorithmic managers will excel in delivering transactional commitments efficiently [47], we argue that the nonfulfillment of transactional promises, as opposed to relational promises, by algorithmic managers will result in lower perceptions of distributive justice. This is because transactional promises are typically characterized by clear, measurable exchanges that align with the strengths of algorithmic systems designed for precision and efficiency [17,48]. When these straightforward expectations are not met, employees might question the fairness of the outcome (distributive justice) more than when relational expectations are not met. Accordingly, we propose the following hypothesis:

**Hypothesis 1:** Employees will experience lower distributive justice when algorithmic managers fail to fulfill transactional promises than when they fail to fulfill relational promises.

### The moderating role of anthropomorphizing algorithmic managers

Algorithmic managers are increasingly being designed with anthropomorphized features to increase their acceptance and effectiveness in the workplace [49]. This design choice is made because humans tend to respond more favorably [50] to systems that appear familiar and relatable [20]. By attributing human-like qualities, organizations aim to reduce the perceived coldness and impersonality that are associated with algorithms [20,21]. Building on prior distinctions in the anthropomorphism literature (e.g., [21,51,52]), we distinguish between two facets of anthropomorphism: *form anthropomorphism* and *behavioral anthropomorphism*. Form anthropomorphism refers to the human-like physical or visual characteristics of an entity (e.g., human-like names, faces, avatars, or body forms), whereas behavioral anthropomorphism concerns human-like social and communicative behaviors, such as conversational language, emotional expressions, or responsiveness. Interestingly, according to anthropomorphism theory [21], when algorithmic systems a) are able to demonstrate conversational abilities and b) have human forms (i.e., are human-like/humanoids), they are viewed more favorably and are endorsed to a greater extent [20,25]. Conversational abilities involve engaging in meaningful, dialog-based interactions rather than simply executing tasks. These abilities represent the “behavioral realism” of algorithmic systems, enabling employees to perceive the algorithmic system as a social entity. This perception can foster greater engagement and enhance positive reactions [52,53]. Human-like forms represent the “form realism” of algorithmic systems and refer to algorithmic systems that have human-like facial expressions or body language. These traits make the system seem more relatable, socially aware, and physically present, thereby enhancing its perceived adaptability and perceived understanding [51,54]. Combining conversational abilities and human-like forms makes algorithmic managers more engaging and less impersonal, fostering positive employee reactions even in challenging interactions [55]. Relatedly, anthropomorphizing algorithmic managers can increase user engagement and potentially soften negative reactions in situations in which algorithmic management systems must deliver critical feedback or enforce decisions [56–58]. Interestingly, the anthropomorphizing of algorithms appears to mitigate negative reactions to decisions made by these systems [55]. More specifically, Yalcin et al. reported that people react less positively to favorable decisions made by an algorithm than to those made by a human, as they struggle to internalize the outcome [55]. However, this effect diminishes when the algorithm is designed with human-like traits, making it more agentic and socially capable.

In this study, we argue that anthropomorphizing algorithmic managers can be particularly beneficial for employees’ perceptions of (in)justice when algorithmic managers fail to fulfill their promises. While anthropomorphism is unlikely to meaningfully change reactions to relational nonfulfillment, because relational obligations hinge on interpersonal qualities (e.g., genuine care, growth support) that employees typically reserve for human managers and do not ascribe to algorithms [17], it can make a notable difference when transactional promises are not fulfilled. Specifically, when algorithmic management is designed with human-like qualities, employees may be more inclined to extend leniency if it fails to fulfill transactional promises, attributing failure to human-like fallibility. This perceived allowance for mistakes can soften negative reactions, as employees may perceive the algorithmic manager not only as a rigid system but also as an entity capable of human-like errors, thereby reducing the perceived injustice resulting from the nonfulfillment. Consequently, anthropomorphizing algorithmic managers may help mitigate harsh judgments in cases where transactional promises are unfulfilled, fostering a more forgiving perception of distributive justice.

Conversely, when an algorithmic manager is perceived as having low anthropomorphism, employees will be stricter in their judgment of failure to meet transactional promises, not because they perceive them as inherently more just but because they are not “allowed” to fail transactional tasks. Since these systems are designed for precision and rule-based execution, any failure in transactional tasks reinforces their perception as rigid and inflexible, as they lack the adaptive, context-sensitive reasoning that humans use to justify errors or exceptions. This aligns with previous work on fairness and justice [59], which suggests that individuals expect stricter adherence to transactional commitments when the entity involved is not perceived as human-like. Accordingly, we argue that when an algorithmic manager appears more mechanical and non-human-like (e.g., machine-like), employees are likely to give less leeway when transactional (as opposed to relational) promises are unfulfilled, as they may see the system as a rigid, inflexible tool rather than an entity capable of human-like errors. Based on the above, we hypothesize the following:

**Hypothesis 2.** The anthropomorphism of algorithmic managers influences the relationship between the type of nonfulfilled promises (transactional versus relational) and employee justice perceptions such that employees will experience lower distributive justice for the nonfulfillment of transactional (as opposed to relational) promises when algorithmic managers are non-anthropomorphized than when they are anthropomorphized.

### The mediating role of algorithmic managers’ perceived rigidity

Extending this rationale, we posit that the perceived rigidity of algorithmic managers mediates the conditional relationship between the type of nonfulfilled promises and perceived justice. *Rigidity* refers to the inflexible, rule-based, and non-context-sensitive characteristics of a system, machine, or technology, such as AI [60]. More specifically, rigidity in algorithmic systems describes a situation where the algorithmic system (i.e., manager) operates on the basis of predefined, rule-based instructions that are unable to account for context, individual preferences, or unexpected variations in human behavior. Rigid systems are perceived as incapable of adjusting their responses or interpreting situations in a flexible manner. This perceived lack of flexibility is often seen as a limitation, especially compared with human managers, who are expected to adapt their responses or actions based on individual circumstances or contextual factors [19,60]. Interestingly, this perceived rigidity can lead to perceptions of unfair treatment when human interactions are involved [19,61].

In this study, we argue that when a non-anthropomorphic algorithmic manager (with the typical, non-human characteristics of algorithm-based tools) fails to fulfill transactional promises, they may be seen as more rigid and trigger, in turn, greater distributive injustice perceptions. This is because transactional promises, such as salary payments or task completions, are clear, measurable commitments with specific expectations [18], and algorithm-based tools are expected to excel in such areas. Therefore, when a non-anthropomorphic algorithmic manager fails to meet these (much expected by said managers) promises, this may come across as a rigidity-related, systemic flaw, highlighting the system’s inability to consider or adapt to contextual factors, respond flexibly to unforeseen situations, or accommodate the nuanced needs of individuals.

In contrast, when the algorithmic manager is highly anthropomorphized, employees may perceive the system as more flexible and capable of understanding the nuances of human interactions. If a highly anthropomorphized algorithmic manager fails to fulfill transactional promises, employees may be more forgiving, attributing the failure to external circumstances rather than a fundamental lack of flexibility or understanding. Consequently, the failure of an anthropomorphic algorithmic manager to meet transactional promises is likely to be perceived less harshly, reducing the perception of rigidity and, in turn, the perceived distributive injustice, compared with when the algorithm is seen as non-anthropomorphic. According to this, we specifically argue that employees will perceive anthropomorphized algorithmic managers as less rigid and non-anthropomorphized ones as more rigid when transactional (as opposed to relational) promises are not fulfilled. Higher rigidity of algorithmic managers, in turn, is related to lower perceptions of distributive justice.

Taken together, our arguments suggest a clear causal chain linking employees’ expectations, their appraisals, and their justice perceptions. Employees typically expect algorithmic managers to be highly competent and precise in handling transactional obligations because these tasks align with algorithms’ data-driven and rule-based capabilities. When these expectations are not met—such as when a transactional promise is not fulfilled—employees are likely to experience a sense of expectancy violation, prompting negative appraisals of the algorithmic manager’s competence and flexibility. This appraisal translates into perceptions of rigidity, as the system is seen as unable to account for contextual nuances or adapt to human needs. Perceived rigidity, in turn, lowers distributive justice judgments by signaling that outcomes are produced through inflexible, impersonal mechanisms. However, when the algorithmic manager is anthropomorphized—displaying human-like intent or social presence—this chain may weaken, as employees attribute more human-like understanding to the system and thus judge its actions less harshly. Based on the above, we propose the following moderated mediation hypothesis:

**Hypothesis 3.** The perceived rigidity of algorithmic managers will mediate the conditional relationship between the type of nonfulfilled promises and employee justice perceptions. Specifically, algorithmic managers who fail to fulfill transactional promises (as opposed to relational promises) will be perceived as less rigid when anthropomorphized and more rigid when non-anthropomorphized. Higher rigidity, in turn, is associated with lower distributive justice.

**Fig 1:**
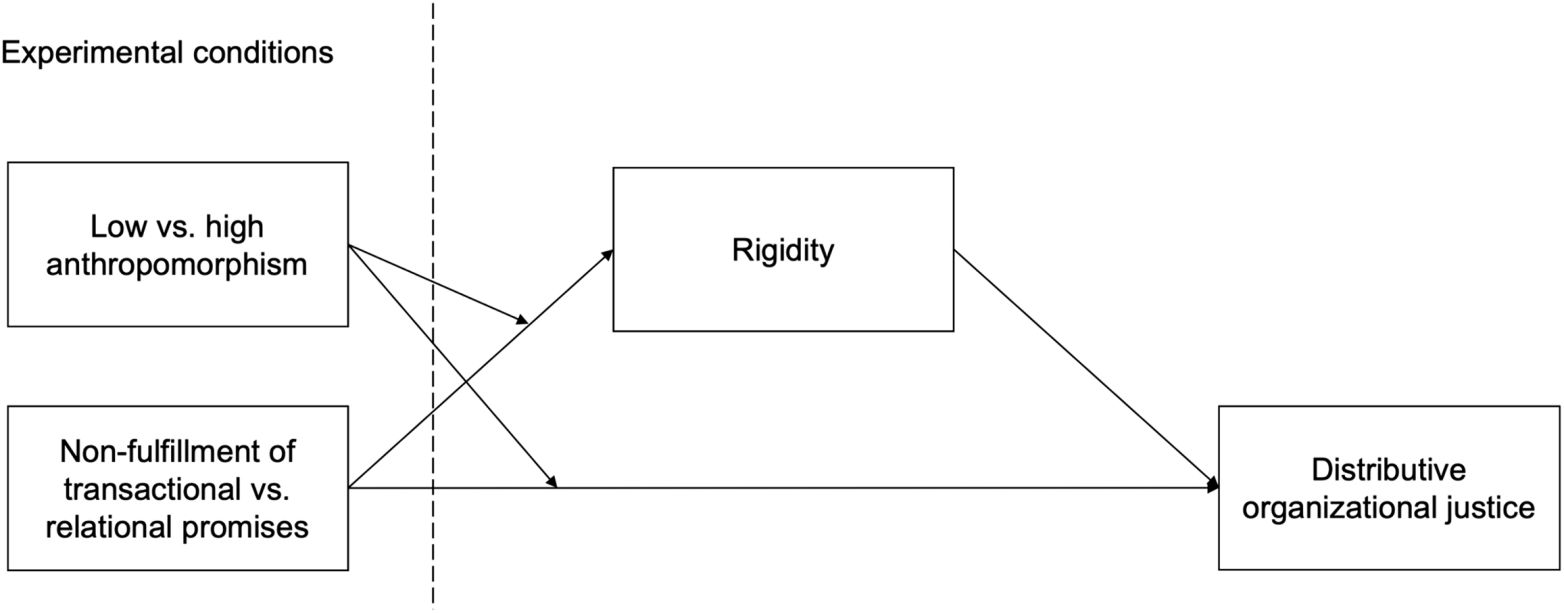
Hypothesized model.

The hypothesized model is illustrated in Fig 1.

### Overview of the present research

We tested our hypotheses in two experiments. Study 1 examined self-referential justice perceptions (participants evaluated distributive justice as if they personally experienced the situation), whereas Study 2 examined broader distributive justice perceptions (participants evaluated justice in terms of how the algorithmic manager would treat employees in general). This distinction allowed Study 2 to capture more generalized justice perceptions of the algorithmic manager, reducing the influence of personal biases that could arise when participants imagine themselves in the scenario [62]. In both experiments, we manipulated the type of nonfulfilled promises and the algorithmic manager’s anthropomorphism in vignettes. Study 1 was a 2 (type of nonfulfilled promises: relational versus transactional) by 2 (anthropomorphism of the algorithmic manager: high versus low) between-subjects experiment aimed at testing Hypotheses 1 and 2. In Study 2, we sought to replicate the findings of Study 1, using alternative vignettes to manipulate the type of nonfulfilled promises, by shifting the focus from career development (Study 1) to broader well-being and personal growth (Study 2). Moreover, in Study 2, we examined the mediating role of the perceived rigidity of algorithmic management (Hypothesis 3), which was not tested in Study 1. Finally, considering that procedural factors such as explanation, voice, and opportunities for recourse can attenuate fairness concerns following negative outcomes [12], we intentionally held such factors constant in our design to isolate employees’ perceptions of outcome fairness in response to unfulfilled promises.

#### Open science and transparency.

The hypotheses for both Studies 1 and 2 were preregistered on the Open Science Framework (OSF). However, the hypotheses in Study 1 differed from those reported in the current manuscript and those preregistered for Study 2. Specifically, in Study 1 (https://osf.io/rwvky/?view_only=dae219fe59ce431a805dda43c3761815), we initially hypothesized that a failure to fulfill relational, as opposed to transactional, promises by algorithmic managers would be associated with lower perceptions of justice, moderated by the level of anthropomorphism of the algorithmic manager. However, the unexpected findings from Study 1 led us to adjust and preregister the revised hypotheses stated in the current manuscript for Study 2 (https://osf.io/48vbw/?view_only=584afb3183234af9815bf1356ea716ba).

Importantly, both studies included additional measures (e.g., work behaviors and attitudes) that are beyond the scope of this paper and are therefore not reported here.

Information regarding ethical, cultural, and scientific considerations specific to inclusivity in global research is included in the Supporting Information (S1 File _Inclusivity_Questionnaire).

## Study 1

### Method

#### Participants.

We recruited 258 participants, all employees from the UK, whose ages ranged from 20--67 years (*M* = 36.76, *SD* = 11.05). All participants were native speakers of English. Participants were recruited online via Prolific (https://www.prolific.com/). Among the participants, 159 (61%) were female, and all worked at least 20 hours per week. Exclusion/inclusion criteria (English language, working at least 20 hours/week) were established via Prolific pre-screening. Fifty-seven participants held tenured positions. A large number held a bachelor’s degree (46.1%) or had completed at least a high school education (30.6%), whereas the remaining participants held higher degrees. Finally, a significant number of the participants worked in the education sector (16.3%), followed by technology (10.5%) and business and finance (9.7%).

According to an a priori power analysis conducted via G*Power for multivariate analysis of variance (MANOVA), our design required a sample size of 200 participants to detect a small effect size of Cohen’s [63] *f*^2^ = 0.06 with a statistical power of 80%. However, our original MANOVA-based a priori power analysis (targeting mean differences) does not directly translate to the smaller interaction effects that are the focus of our hypotheses. Accordingly, we also conducted a sensitivity analysis aligned with the focal model. *G*Power’s *linear multiple regression was used: Fixed model, R² increase* (test family: F tests), with *N* = 258, α = 0.05, tested predictor = 1 (the interaction), and total predictors = 5; the smallest detectable incremental effect for the interaction was *f*^2^ = 0.06 at 95% power.

The data collection started on 5 September 2024 and was completed on 12 September 2024. Participation lasted approximately seven minutes, and participants were compensated €1 (≈£0.87, which corresponds to €8.57 per hour ≈ £7.45/hour) for their time. Prior to the data collection, written ethical approval was granted by the Scientific and Ethical Review Board of the Faculty of Behavioral and Social Sciences of the University of Groningen, the Netherlands. (Nr of ethics approval is: PSY-2324-S-0402). The board confirmed that the research complied with institutional ethical guidelines. All participants provided their active informed consent prior to participation.

#### Experimental procedure and manipulations.

This study utilized a 2 (type of nonfulfilled promises: relational versus transactional) × 2 (anthropomorphism of the algorithmic manager: high versus low) between-subjects experimental design. We used Qualtrics to program the study and ensured that the participants were randomly assigned to any of the experimental conditions presented below. Importantly, throughout the manuscript, we use the term “*algorithmic manager*” to refer to AI-driven systems that make or support managerial decisions. In the experimental materials, however, we refer to a “*robot manager*” and provide robot-like images to ensure clarity and vividness for participants, as previous work shows that abstract algorithmic systems are difficult to mentally visualize in vignette settings. Importantly, the robot manager in our vignettes is presented as the managerial decision-maker and thus serves as the concrete instantiation of an algorithmic manager for the purposes of our manipulations.

The instructions invited participants to immerse themselves in the role of an employee working in a large company named “Beta Management Company” and being supervised by a robot manager instead of a human manager. We manipulated the anthropomorphism of the robot manager based on Tomprou and Lee [17], Wang et al. [64] and Yam et al. [50]. Importantly, we manipulated both *form anthropomorphism* and *behavioral anthropomorphic* facets in combination to create high- versus low anthropomorphism conditions. Specifically, the high-anthromorphism condition featured an algorithmic manager with a human-like name and visual representation (form anthropomorphism) that communicated via conversational and socially responsive language (behavioral anthropomorphism), whereas the low-anthropomorphism condition excluded these features. Our manipulations were as follows:

**Low Anthropomorphism Condition.** In this condition, participants were introduced to a robot manager named “Robo3000.” Robo3000’s role was described as providing instructions, requesting updates, and being accessible only through a chat tool, with no face-to-face interactions. Key details emphasized its non-anthropomorphic nature: Robo3000 was located in the IT department, operated 24/7 without breaks (except for one day of monthly maintenance), and it was introduced simply as “Robo3000” without a human-like name or characteristics. This setup was designed to present Robo3000 as a tool-like rather than a human-like robot. To reinforce this manipulation, after the participants were introduced to their robot manager, they were specifically asked to think of Robo3000 as a robot rather than a real person and to list ideas about its functioning at work, emphasizing its nonhuman qualities: “*Please write down two ideas that come to mind about how Robo3000 functions at work, considering that it is a robot rather than a real person*”. Additionally, participants were shown an image of their robot manager, depicting it as a purely mechanical entity.

**High Anthropomorphism Condition.** The participants in this condition were introduced to a robot manager who had human-like characteristics such as a human-like name, gender, and approachable demeanor—either “Lily Adams” (female version) or “Noah Adams” (male version). The robot manager introduced itself directly to participants, saying, “Hello, I am [Lily/Noah] Adams, your new manager and companion at work.” The description emphasized physical proximity (e.g., “My office is on the same floor as yours”), human-like interaction norms (e.g., “…available during normal office hours, just like everyone else”), and direct communication, where employees could approach the robot manager for clarifications or help. To reinforce this manipulation, after being introduced to their robot manager, participants were specifically asked to think of their manager as a person rather than a robot and to list qualities they thought Lily/Noah might exhibit at work: “*Please try to think of her/him as a person rather than a robot and write down two ideas that come to mind about the type of person you think Lily/Noah Adams is at work*”. Furthermore, participants were shown an image of their robot manager, depicted as a human-like, anthropomorphic figure with either female or male characteristics. The images of the anthropomorphic managers were generated via advanced AI-based image creation tools (Midjourney and DALL·E 3) with carefully crafted prompts to ensure neutrality and consistency across stimuli. Specifically, we instructed the AI to produce male and female manager images that were comparable and neutral in terms of attractiveness, age, and perceived competence, while differing only in terms of human likeness to reflect the anthropomorphism manipulation. This approach minimized potential confounding factors related to visual appearance or social desirability cues. Gender versions of the algorithmic managers were randomized between subjects. The full vignettes and images are presented in the Online Supplemental Material (see supplemental material; S2 File).

We also manipulated the robot manager’s failure to fulfill relational versus transactional promises in vignettes, inspired by Tomprou and Lee [17]. In the transactional condition, the robot manager emphasized immediate, task-oriented, and tangible rewards consistent with a short-term, exchange-based approach. Conversely, in the relational condition, the robot manager focused on more long-lasting and relational aspects, highlighting the robot manager’s interest in fostering a meaningful relationship [65]. Specifically, the manipulations were as follows:

**Nonfulfillment of Transactional Promises.** Participants were led to believe that their robot manager had made specific promises regarding their compensation and performance incentives during contract negotiations. These promises included assurances of consistent salary benchmarking, annual salary raises to maintain their standard of living, and regular bonuses based on performance targets. After two years of employment, however, participants were informed that these promises had not been fulfilled, as they had not received any bonuses, their salaries had not been adjusted for inflation, and they were denied a promised pay raise despite excellent performance evaluations.

**Nonfulfillment of Relational Promises.** The participants read a scenario in which the robot manager had committed to supporting employees’ professional growth and personal well-being. Participants were told that they could expect access to specialized training workshops, opportunities for professional networking, and assistance with personal issues. However, after two years, their requests for training were denied, they had no opportunities for networking, and their requests for personal time off were unapproved (see S2 File for complete vignettes).

All participants first completed the manipulation checks immediately after reading the vignettes, followed by the perceived distributive justice measure.

### Measures

#### Manipulation checks.

*Anthropomorphism of the Robot Manager.* The manipulation check items for the perceived anthropomorphism of the robot manager comprised two items measured on a 7-point Likert scale (1 = *not at all*, 7 = *to a great extent*). The items were “Did the robot manager’s appearance lead you to anthropomorphize it (= perceive it as more human-like) by attributing human-like thoughts, feelings, or intentions to it/him/her?”, and “How interactive and engaging did your collaboration with your robot manager feel?”. A mean score was computed for these two items, yielding a Cronbach’s alpha of *α* = .72.

*Type of Nonfulfilled Promises.* We used one binary manipulation check item for the type of unfulfilled promises by the robot manager (“Would you characterize the nature of the promises made by your Robot Manager: 1 = *relational* [e.g., promoting mutual respect and engaging interactions], 7 = *transactional* [e.g., promoting instrumental relations and focusing on task completion], inspired by Tomprou and Lee [17].

**Perceived Distributive Justice** We measured distributive justice using Colquitt’s [24] distributive justice sub-measure, which we adapted to the specifics of this study. Participants were first instructed to imagine their collaboration with their robot manager, as described in the scenario they read, and answer questions such as if this collaboration had happened in real life. *Distributive justice* was assessed with a 4-item scale, measuring perceptions of outcome fairness in relation to effort and contribution [66]. The items included “If what you read in the above scenario had happened in real life, to what extent would you feel that... …the outcomes you received from your robot manager reflect the effort you have put into your work” (*α* = .95).

**Control Variables.** We controlled for participants’ tenure position (Do you have a tenured position?; 1 = *yes*, 2 = *no* and general attitudes toward AI, as both variables have been found to influence perceptions of algorithm use at work [67,68]). We measured attitudes toward AI with the four-item scale of Grassini [69]. The respondents rated their agreement with each statement on a 10-point scale ranging from 1 = *not at all* to 10 = *completely agree* (*α* = .91).

### Results

#### Main analysis.

SPSS was used for all the statistical tests. To test the moderation model, we conducted a moderation analysis (Model 1) via the PROCESS macro [70]. We applied 5,000 bootstrap resamples with bias-corrected confidence intervals to ensure robust estimation of the interaction effect and enhance the reliability of our findings. When testing the model for normality, homoscedasticity, and multicollinearity, we found that these assumptions of regression analysis were met. Detailed diagnostics and outputs are provided in the Supporting Information (S3 File).

#### Manipulation checks.

We conducted a 2 (type of nonfulfilled promises: relational versus transactional) × 2 (algorithmic manager’s anthropomorphism: high versus low) MANOVA to test whether the manipulation of the type of nonfulfilled promises and anthropomorphism influenced the respective manipulation check items as intended. As expected, the anthropomorphism of the manager significantly influenced the respective manipulation check measure, showing that participants experienced the algorithmic manager as more anthropomorphic in the high-anthropomorphism condition (*M* = 3.57, *SD* = 1.37) than in the low-anthropomorphism condition (*M* = 2.30, *SD* = 1.24), *F*_(1,254)_ = 60.25, *p* < .001, *η*^2^ = .19. Moreover, the manipulation of the type of nonfulfilled promises had a marginally significant effect on the respective manipulation check item, showing that participants perceived the promises being nonfulfilled as more transactional in the transactional condition (*M* = 5.51, *SD* = 1.74) than in the relational condition (*M* = 5.11, *SD* = 1.73), *F*_(1,254)_ = 3.38, *p* = .06, *η*^2^ = .01. (Considering the marginally significant effect, we conducted additional sensitivity analyses testing the robustness of the manipulation checks; Results showed that key coefficients remained relatively stable. The results can be found in S4 File). Importantly, the anthropomorphization of the manager did not significantly influence the manipulation check item assessing the type of unfulfilled promises. Similarly, the type of unfulfilled promises did not significantly influence the manipulation check for perceived anthropomorphism of the manager. Moreover, no interaction effects were significant (*F*s < 1). These results indicate that the manipulations generally operated as intended, although the marginally significant effect observed for the manipulation of the type of nonfulfilled promises represents a limitation.

#### Hypothesis testing.

**Fig 2:**
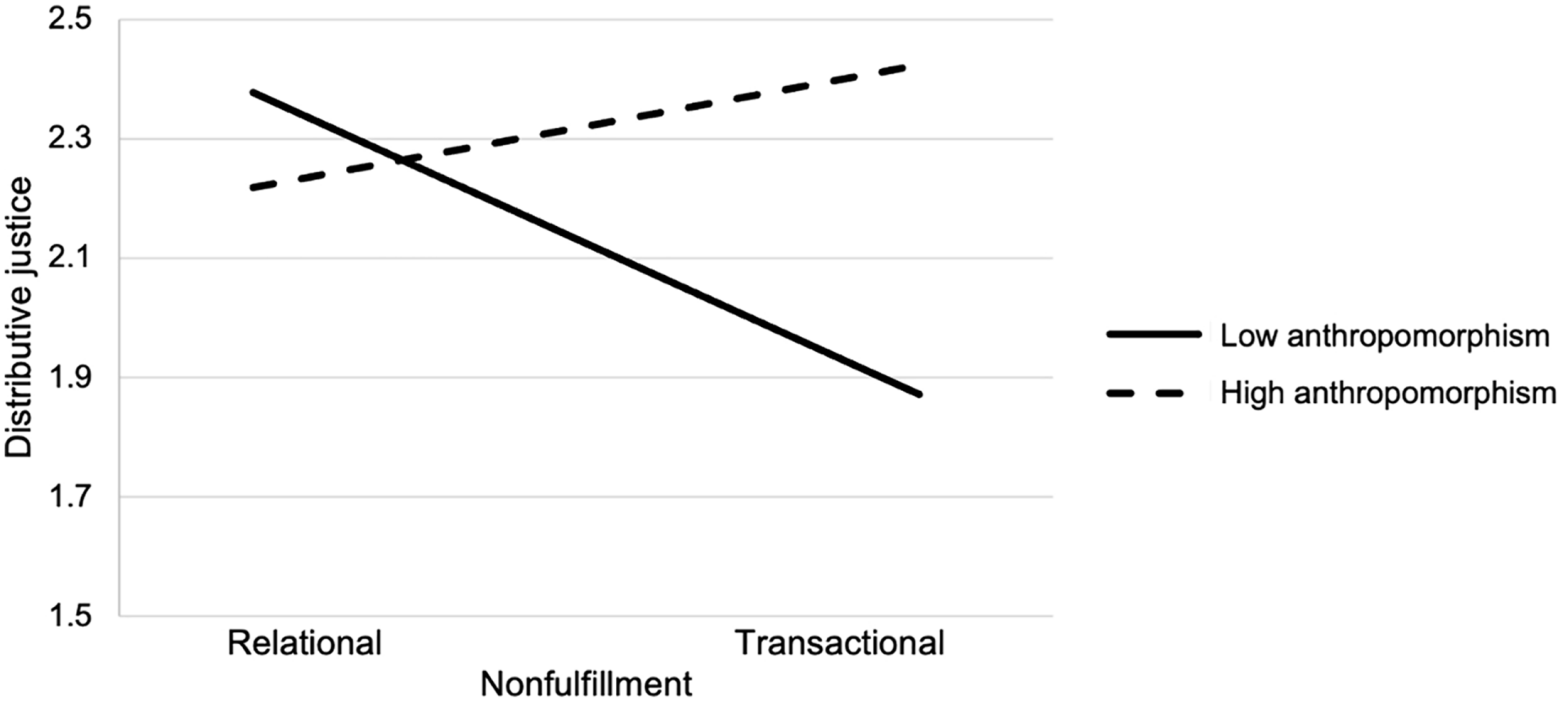
Anthropomorphism Moderates the Relationship between Nonfulfillment of Transactional versus Relational Promises and Perceived Distributive Justice. *Note.* Perceived distributive justice was measured on a 7-point scale (1 = *to a very small extent*, 7 = *to a very large extent*). The y-axis has been truncated to emphasize the relevant range of values.

Descriptive statistics and correlations between the study variables can be found in Table 1. Table 2 reports descriptive statistics per cell. In the analyses, we coded nonfulfillment as follows: 0 = relational promises; 1 = transactional promises. Anthropomorphism was coded as follows: 0 = low; 1 = high. Continuous predictors were mean centered. In Hypothesis 1, we suggested that the manager’s failure to fulfill transactional, as opposed to relational, promises is negatively related to perceptions of distributive justice. Indeed, nonfulfillment of transactional (as opposed to relational) promises was significantly and negatively related to distributive justice (*B* = −.51, *p* < . 05; Table 3). Therefore, Hypothesis 1 was supported. Hypothesis 2 stated that anthropomorphism moderates the relationship between nonfulfillment and distributive justice. The results show that anthropomorphism indeed moderates the relationship between nonfulfillment and distributive justice (*B* = .71, *p* < .01; Table 3). With low anthropomorphism, nonfulfillment of transactional (rather than relational) promises is seen as less distributively just than with high anthropomorphism (Fig 2). Because the direction of the moderated relationships is as hypothesized, we conclude that Hypothesis 2 is supported.

**Table 1:** Descriptive statistics and correlations of the study variables (studies 1 and 2).

	Study 1		Study 2						
	*M*	*SD*	*M*	*SD*	1	2	3	4	5
1 AI attitude	5.58	1.57	7.41	1.82		−.07	−.07	.07	.00
2 Tenured position	1.78	.42	1.79	.41	−.08		−.04	.05	−.05
3 Nonfulfillment					−.06	−.01		.00	−.05
4 Anthropomorphism					−.03	−.02	−.00		.06
5 Distributive justice	2.22	1.40	3.39	1.63	.25***	.02	−.07	.06	
6 Rigidity			5.85	1.50	−.00	.01	.11	.06	−.18**

*Notes*. Nonfulfillment was coded as follows: 0 = *Relational promises*; 1 = *Transactional promises*. Anthropomorphism was coded as follows: 0 = *Low*; 1 = *High*. Correlations of Study 1 are above the diagonal, and the correlations of Study 2 are below the diagonal.

**p < .01. ***p < .001.

**Table 2:** Descriptive statistics per cell of the dependent variable distributive justice (study 1).

	Transactional promises			Relational promises		
Anthropomorphism condition	*N*	*M*	*SD*	*N*	*M*	*SD*
Low	63	1.88	1.32	65	2.38	1.36
High	64	2.42	1.65	66	2.21	1.19

**Table 3:** Relationship between Nonfulfillment of Promises (Transactional versus Relational) and Distributive Justice as Moderated by Anthropomorphism (Study 1).

	Model with control variables				Model with moderation					
	*B*(*SE*)	*β*	*p*	*CI*	*B*(*SE*)	*β*	*p*	*CI*	*sr*	*f* ^*2*^
Constant	2.56(.52)	−.05		1.54,3.58	2.83(.54)		.00	1.76,3.89		
AI attitude	−.00(.06)	−.00	.95	−.12,.11	−.01(.06)	−.01	.83	−.12,.10	−.01	.000
Tenured position	−.18(.21)	−.05	.40	−.60,.24	−.21(.21)	−.06	.31	−.63,.20	−.06	.004
Nonfulfillment					−.51(.25)	−.18	.04	−.99,-.02	−.13	.017
Anthropomorphism					−.16(.24)	−.06	.52	−.64,.32	−.04	.002
Nonfulfillment* anthropomorphism					.71(.35)	.22	.04	.03,1.40	.13	.017
R^2^	.00		.70		.03		.24			
∆R^2^					.03					

*Note.*.02 ≤ *f*^*2*^ <.15 indicates a small effect size; sr=semipartial r.

Notably, as a robustness check, we ran the same analysis with the gender of the robot manager as an additional control variable, and the results were very similar to those reported. The output can be found in the study project in the Open Science Framework.

### Discussion

Study 1 aimed to examine how nonfulfillment of transactional versus relational promises by algorithmic managers influences employees’ perceptions of distributive justice, considering the moderating role of algorithmic managers’ anthropomorphism. In line with Hypothesis 1, the type of nonfulfillment had a significant effect on distributive justice, showing that employees perceive the nonfulfillment of transactional promises as opposed to relational promises as more distributively unfair. Moreover, consistent with Hypothesis 2, the nonfulfillment of transactional promises, as opposed to relational promises, was associated with lower perceptions of distributive justice when the algorithmic manager was not anthropomorphized. However, justice perceptions did not significantly differ across transactional and relational conditions when the algorithmic manager was anthropomorphized. This finding indicates that employees are more sensitive to nonfulfillment of transactional promises when algorithmic managers appear less human-like, likely because such systems—when they come across as robots rather than human-like—are expected to excel in fulfilling standardized, transactional tasks [71].

Overall, the findings partially support Hypothesis 2, highlighting the role of the anthropomorphism of algorithmic systems in shaping distributive justice perceptions related to the nonfulfillment of transactional promises. Study 1 did not test the mediating role of the perceived rigidity of algorithmic management, which was done in Study 2.

## Study 2

### Method

#### Participants.

We recruited 248 participants, all employees. Participants were recruited online via Prolific. Among the participants, 45% came from the UK, whereas 54.8% came from the US. All participants were native or fluent speakers of English. Their ages ranged from 22 to 60 years (*M* = 37.12, *SD* = 9.46). Among the participants, 141 (56.9%) were female, and all worked at least 20 hours per week. Fifty-one participants held tenured positions. Half of the participants held a bachelor’s degree (50%), forty-nine participants (19.8%) had completed high school, and the remaining participants held higher degrees. A significant number of the participants worked in the technology sector (19.8%), followed by business and finance (14.9%) and education (14.1%). Similar Prolific pre-screeners as in Study 1 were used in Study 2 as well.

We estimated power for the conditional indirect effect at each anthropomorphism level via the Schoemann–Boulton–Short Monte Carlo app (One Mediator; Input: Correlations derived from our conditional path estimates; 20,000 draws/rep, 1,000 reps, 95% CIs). When *N* = 248, the estimated power to detect the indirect effect was 0.54 for low anthropomorphism and 0.40 for high anthropomorphism.

The data collection started on 10 October 2024 and was completed on 20 October 2024. Similar to Study 1, we obtained ethics approval and the written informed consent of the participants prior to the data collection. Participation lasted approximately seven minutes and participants were compensated €1 (≈£0.87, which corresponds to €8.57 per hour ≈ £7.45/hour).

#### Experimental procedure and manipulations.

Similar to Study 1, a 2 (type of nonfulfilled promises: relational versus transactional) × 2 (anthropomorphism of the robot manager: high versus low) between-subjects experimental design was used for this study. Participants were randomly assigned to one of four conditions. We manipulated the anthropomorphism of the robot manager similarly to Study 1. The manipulation of the type of nonfulfilled promises was similar to that in Study 1, but the specific promises differed, allowing us to test whether the findings of Study 1 would replicate with alternative transactional and relational promises. More specifically, in the transactional condition, the robot manager focused on productivity-related promises, emphasizing task-oriented, tangible rewards, including milestone-based bonuses, overtime compensation, and merit-based salary increases. The participants were later informed that these promised incentives were not fulfilled despite their consistent performance. In contrast, the relational condition in Study 2 portrayed the robot manager as committed to participants’ professional development and well-being, offering individualized mentorship, growth opportunities through workshops, and holistic wellness initiatives. However, participants learned that these supportive, development-focused promises were unfulfilled, as no mentorship program, workshops, or wellness support was provided. The vignettes are available in the online supplemental material in S2.

The participants first completed the manipulation checks immediately after reading the vignettes, followed by the perceived rigidity and distributive justice measures.

### Measures

**Manipulation Checks.** We used the same manipulation check items for the type of nonfulfilled promises (one item) and the perceived anthropomorphism of the robot manager (two items: *α* = .77) as in Study 1.

**Perceived Rigidity of the Robot Manager.** We measured the perceived rigidity of the robot manager with one item: “How rigid do you think your Robot Manager would be in following company rules and policies, even when flexibility might be needed?” (1 *= not at all rigid*, 7 = *very rigid*).

**Perceived Distributive Justice** Distributive justice was measured based on the basis of Colquitt [24]. However, the items were further adapted to the specifics of the vignettes. The participants were instructed to bring back to mind their collaboration with their robot manager and indicate their opinion about how such robot managers would act when used at work. The *distributive justice* scale included four items in total (*α* = .91). A sample item was “I believe that the rewards and recognition employees would receive from such a robot manager would be fair and based on employee performance”. Like in Study 1, we used a 7-point Likert scale to measure organizational justice (1 = *to a very small extent* 7 = *to a very large extent*). The complete measures can be found in the online supplemental material in S2 File.

**Control Variables.** We used the same control variables as in Study 1. The reliability of the general attitudes toward AI was.92.

### Results

#### Manipulation checks.

Similar to Study 1, we conducted a 2 (type of nonfulfilled promises: relational versus transactional) × 2 (algorithmic manager’s anthropomorphism: high versus low) MANOVA to test whether our manipulations worked as intended. As expected, the anthropomorphism of the manager significantly influenced the respective manipulation check measure, showing that participants experienced the algorithmic manager as more anthropomorphic in the high-anthropomorphism condition (*M* = 3.67, *SD* = 1.61) than in the low-anthropomorphism condition (*M* = 2.50, *SD* = 1.47), *F*_(1,244)_ = 36.32, *p* < .001, *η*^2^ = .13. Moreover, the manipulation of the type of nonfulfilled promises had a significant effect on the respective manipulation check item, showing that participants perceived the nonfulfilled promises as more transactional in the transactional condition (*M* = 6.30 *SD* = 1.08) than in the relational condition (*M* = 4.98, *SD* = 1.94), *F*_(1,244)_ = 44.41, *p* < .001, *η*^2^ = .15. As in Study 1, the anthropomorphization of the manager did not significantly influence the manipulation check item assessing the type of unfulfilled promises. Similarly, the type of unfulfilled promises did not significantly influence the manipulation check for perceived anthropomorphism of the manager. Moreover, no interaction effects were significant (all *F*’s < 1). We conclude that our manipulations worked as intended.

### Hypothesis Testing

The descriptive statistics and correlations among the study variables are reported in Table 1. Table 4 reports descriptive statistics per cell. To test the model, we used a moderated mediation model (Model 8) in the PROCESS macro [70] with 5,000 bootstrap resamples and bias-corrected confidence intervals to estimate indirect effects. This approach enhances the robustness and reliability of our findings by accounting for the sampling distribution of the indirect effects. As in Study 1, the assumptions of normality, homoscedasticity, and multicollinearity were met. Detailed diagnostics and outputs are provided in the Supporting Information (S5 File). Like in Study 1, in the analyses, we coded nonfulfillment as follows: 0 = relational promises; 1 = transactional promises. Anthropomorphism was coded as follows: 0 = low; 1 = high. Continuous predictors were mean centered.

**Table 4:** Descriptive statistics per cell of the dependent variable distributive justice and the mediator rigidity (study 2).

	Transactional promises					Relational promises				
		Distributive justice		Rigidity			Distributive justice		Rigidity	
**Anthropomorphism**	***N***	***M***	***SD***	***M***	***SD***	***N***	***M***	***SD***	***M***	***SD***
Low	63	3.20	1.73	6.13	1.33	59	3.40	1.74	5.36	1.97
High	65	3.35	1.65	5.88	1.45	61	3.62	1.39	6.00	1.07

**Fig 3:**
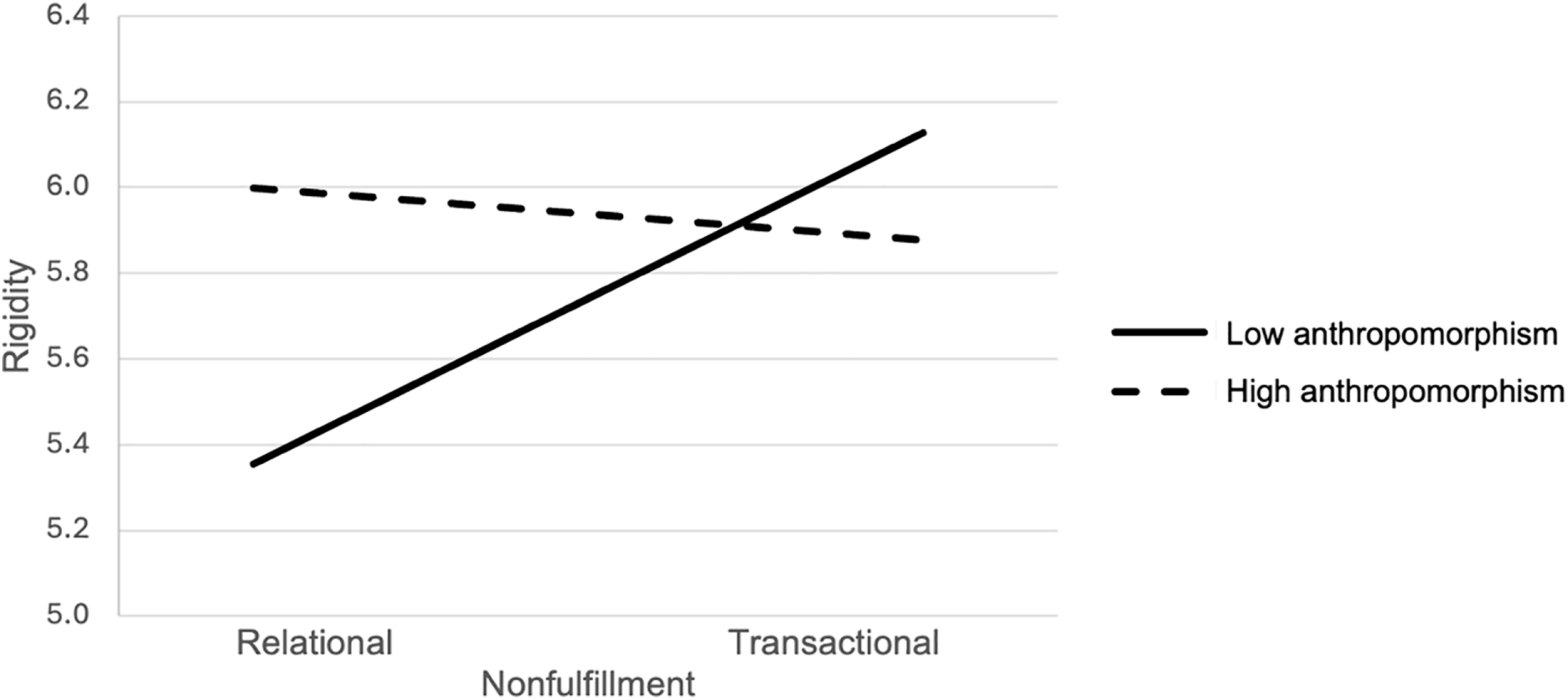
Anthropomorphism Moderates the Relationship between Nonfulfillment of Transactional versus Relational Promises and Perceived Rigidity of the Algorithmic Manager. *Note.* Perceived rigidity was measured on a 7-point scale (1 *= not at all rigid,* 7 = *very rigid*). The y-axis has been truncated to emphasize the relevant range of values.

In testing Hypothesis 1, which proposes a negative relationship between nonfulfillment of transactional (as opposed to relational) promises and distributive justice, nonfulfillment was not related to distributive justice, thus, we failed to find support for Hypothesis 1 (*B* = −.02, *ns*, Table 5). Hypothesis 2 focused on the direct interaction effect between nonfulfillment and anthropomorphism on distributive justice. Unexpectedly, no direct moderation effect occurred (*B* = −.21, *ns;* Table 5); thus, Hypothesis 2 was not supported. To test Hypothesis 3, we investigated the relationship between the nonfulfillment of promises and distributive justice mediated by the rigidity of algorithmic managers and moderated by anthropomorphism. As expected, the type of nonfulfillment (transactional versus relational) and anthropomorphism interact to significantly predict the perceived rigidity of algorithmic managers (*B* = −.89, *p* < .05, Table 5): Under the condition of low anthropomorphism, nonfulfillment of transactional promises as opposed to relational promises was related to higher rigidity (*B* = .77, *p* < .01, 95% CI [.24, 1.30]), whereas no mean difference occurred under the high anthropomorphism condition (*B* = −.12, *ns*, 95% CI [−.65,.40], Fig 3). The mediator rigidity was in turn significantly and negatively related to distributive justice (*B* = −.20, *p* < .01; Table 5). The moderated mediation was significant under the low-anthropomorphism condition (*Est.* = −.15, BootCI [−.33, −.01], Tables 6 and 7). Therefore, Hypothesis 3 was supported.

**Table 5:** Relationship between Nonfulfillment of Promises (Transactional versus Relational) and Distributive Justice as Moderated by Anthropomorphism and Mediated by Rigidity (Study 2).

	Rigidity										Distributive Justice									
	Control variables				Moderation						Control variables				Moderated mediation					
	*B* (*SE*)	*β*	*p*	*CI*	*B* (*SE*)	*β*	*p*	*CI*	*sr*	*f* ^*2*^	*B* (*SE*)	*β*	*p*	*CI*	*B* (*SE*)	*β*	*p*	*CI*	*sr*	*f* ^*2*^
Constant	5.80 (.61)		.00	4.60,7.00	5.32 (.63)		.00	4.08,6.57			1.40 (.64)		.03	.13,2.66	2.44 (.75)		.00	.96,3.93		
AI attitude	−.00 (.05)	−.00	.99	−.11,.10	.00 (.05)	.00	.95	−.10,.11	.00	.00	.23 (.06)	.26	.00	.12,.34	.23 (.06)	.25	.00	.12,.34	.25	.07
Tenured position	.03 (.24)	.01	.90	−.44,.50	.00 (.24)	.00	.99	−.46,.47	.00	.00	.17 (.25)	.04	.50	−.32,.66	.17 (.25)	.04	.50	−.32,.66	.04	.00
Nonfulfillment					.77 (.27)	.26	.01	.24,1.30	.18	.03					−.02 (.29)	−.01	.95	−.59,.55	−.00	.00
Anthropomorphism					.64 (.27)	.22	.02	.11,1.18	.15	.02					.35 (.29)	.11	.22	−.22,.92	.07	.01
Nonfulfillment* anthropomorphism					−.89 (.38)	−.26	.02	−1.64,-.15	−.15	.02					−.21 (.40)	−.06	.61	−1.00,.59	−.03	.00
Rigidity															−.20 (.07)	−.18	.00	−.33,-.07	−.18	.04
R^2^	.00		.99		.04		.10				.07		.00		.10		.00			
∆R^2^					.04										.03					

*Note.*.02 ≤ *f*^*2*^ <.15 indicates a small effect size; sr=semipartial r.

**Table 6:** Effects of moderation and moderated mediation (study 2).

Path	Values of moderator anthropomorphism	*B*(*SE*)	LLCI	ULCI
Nonfulfillment → Rigidity	Low	.77(.27)**	.2390	1.3040
	High	−.12(27)	−.6470	.4032
Nonfulfillment → Distributive justice	Low	−.02(.29)	−.5861	.5504
	High	−.22(.28)	−.7752	.3276
		*B(BootSE)*	BootLLCI	BootULCI
Nonfulfillment → Rigidity → Distributive justice	Low	−.15(.08)	−.3888	−.0366
	High	.02(.05)	−.0463	.1642

*Notes*. Nonfulfillment was coded as follows: 0 = *Relational promises*; 1 = *Transactional promises*. Anthropomorphism was coded as follows: 0 = *Low*; 1 = *High*

**p < .01.

**Table 7:** Index of moderated mediation (study 2).

Dependent variable	Index	BootSE	BootLLCI	BootULCI
Distributive justice	.1772	.1040	.0321	.4730

Similar to Study 1, as a robustness check, we ran the same analysis with the gender of the robot manager as an additional control variable, and the results were very similar to those reported. The output can be found in the study project in the Open Science Framework.

### Discussion

Unlike Study 1, Study 2 revealed no significant main effect of the type of non-fulfilled promises on distributive justice (Hypothesis 1; not supported). This might be because employees may not perceive promise nonfulfillment by algorithmic managers as sharply differentiated between transactional and relational, as algorithmic managers are tools rather than entities with moral agency. Moreover, in contrast to Hypothesis 2, we found no direct interaction effects between the type of promise nonfulfillment and anthropomorphism on distributive justice. However, in line with Hypothesis 3, the nonfulfillment of transactional (as opposed to relational) promises was associated with higher perceptions of algorithmic manager rigidity, which was subsequently related to lower distributive justice perceptions *only* when the algorithmic manager was not anthropomorphized. Like in Study 2, justice perceptions did not significantly differ across transactional and relational conditions when the algorithmic manager was anthropomorphized. Taken together, these findings suggest that employees expect low-anthropomorphic algorithmic managers to efficiently fulfill transactional promises, and when these expectations are unfulfilled, algorithmic managers are perceived as inflexible/rigid and, in turn, less distributively fair.

## General discussion

Various managerial roles within workplaces are being given to algorithmic management, a shift that significantly impacts employees’ experiences [1], especially with respect to perceptions of justice [3]. The present research examined how employees evaluate the distributive justice of algorithmic managers who fail to fulfill relational versus transactional promises and how these evaluations are shaped by the manager’s level of anthropomorphism. Across two experiments, we found both replication and divergence in the tested relationships (Hypothesis 1–Hypothesis 3). Specifically, the main effect predicted in Hypothesis 1—that nonfulfillment of transactional (versus relational) promises would lead to lower distributive justice—was supported in Study 1 but not replicated in Study 2. The moderating effect of anthropomorphism (Hypothesis 2) emerged in Study 1 but not in Study 2, whereas the moderated mediation effect proposed in Hypothesis 3 was tested (and supported) only in Study 2. These mixed results indicate that the observed effects are not fully robust and should be interpreted cautiously.

A key reason for the divergent findings across studies likely lies in the different perspectives that were activated when assessing distributive justice. In Study 1, participants assessed distributive justice from a self-referential standpoint, evaluating how just the outcomes felt to them personally. In Study 2, however, participants adopted a more generalized, system-level perspective, assessing how just an algorithmic manager would treat employees in general. This shift in reference frame provides a plausible explanation for the nonreplication of the direct interaction effects observed in Study 1. Self-focused justice assessment may elicit stronger affective reactions, which may amplify differences between relational and transactional promise nonfulfillment. In contrast, generalized justice assessment may evoke more detached, norm-based judgments, which may dilute or obscure these distinctions. Thus, the absence of direct effects in Study 2 suggests that the theoretical predictions may apply primarily when individuals evaluate their own treatment rather than the treatment of employees more broadly.

Another key difference that may have influenced our findings is the way relational and transactional promise unfulfillment was manipulated across the two studies. In Study 1, transactional promises were framed primarily around salary-related commitments, whereas relational promises focused on career development. In contrast, Study 2 shifted the emphasis to task- and performance-related transactional promises, whereas relational promises were framed more broadly in terms of well-being and personal growth. This variation in framing may have influenced how participants perceived and distinguished between the two types of promises, potentially contributing to differences in our findings.

Taken together, these methodological differences suggest that the proposed effects are context-dependent and that boundary conditions related to justice evaluation perspective and promise framing should be incorporated into the theoretical framework to more accurately determine when and why anthropomorphism of algorithms shapes justice perceptions.

### Theoretical implications

This study makes three significant theoretical contributions. First, it builds on prior research on algorithmic management (e.g., [1]) and psychological contracts (e.g., [17,25]) by examining how employees perceive justice when algorithmic management fails to fulfill relational versus transactional promises. The literature on psychological contract breach often associates relational inducements with human managers (e.g., [17,25]), whereas transactional inducements are typically associated with both algorithmic and human managers. By examining how algorithmic managers’ failure to fulfill relational versus transactional promises influences perceptions of distributive justice, this study enhances our understanding of employees’ expectations regarding these managers’ ability to handle distinct types of commitments. This research highlights how employees differentiate between relational promises, which emphasize personal support, growth opportunities, and a sense of care [35], and transactional promises, which focus on efficiency and task completion, shedding light on the way in which algorithmic managers are evaluated based on the nature of their promises. This is crucial given the growing role of algorithmic management in various organizational tasks, from rewarding to decision-making and the emotional support of employees [1].

Second, the study draws on anthropomorphism theory [20,21] to explore how human-like qualities in algorithmic management systems can mitigate the negative effects of unfulfilled promises. Our findings demonstrate that employees perceive lower distributive and interactional justice when their non-anthropomorphized managers fail to fulfill transactional promises than when they fail to fulfill relational promises. This suggests that employees are more likely to view non-anthropomorphized managers as rigid, leading to harsher judgments of fairness. These results expand the anthropomorphism theory and algorithmic management literature by showing how the human-like traits of algorithmic managers can influence perceptions of justice [22].

The above findings support the proposed causal chain linking employees’ expectations, appraisals, and justice perceptions. Across both studies, nonfulfillment of expected competence and flexibility led employees to appraise the algorithmic manager as rigid, which subsequently reduced perceived distributive justice. This pattern provides integrative evidence for our theorized mechanism—linking unmet psychological contracts, perceptions of anthropomorphism, and fairness judgments—while complementing the broader contributions to the psychological contract and anthropomorphism theories discussed above.

Third, our study contributes to the organizational justice and AI literature [3,11] by highlighting the unique challenges employees face when algorithmic managers fail to fulfill promises. By investigating the nonfulfillment of both relational and transactional promises, this study deepens our understanding of how unfulfilled promises affect employees’ perceptions of distributive justice. These findings highlight the importance of considering both the type of promises nonfulfillment and the anthropomorphic characteristics of algorithmic managers to better understand and address fairness concerns in algorithmic management [25].

Taken together, while prior research has examined anthropomorphism and justice perceptions separately, this study integrates these literatures within the novel context of algorithmic management and psychological contract theory. By focusing on distributive justice following the nonfulfillment of promises, we extend existing models of organizational justice to a technology-mediated context where managerial agency is algorithmic rather than human. Furthermore, by identifying perceived rigidity as a key appraisal mechanism, we advance the understanding of how and why anthropomorphism influences fairness judgments, thereby refining theories of both anthropomorphism and justice in human–AI interactions.

### Practical implications

From a practical standpoint, this research offers valuable insights for organizations increasingly relying on algorithmic management. Since employees tend to view anthropomorphized algorithmic managers as less rigid when they fail to fulfill their (transactional) commitments, organizations might consider integrating human-like characteristics into the design and communication of algorithmic management systems to improve perceptions of justice, especially in situations where psychological contract breaches occur. When algorithmic managers are perceived as human-like, employees may be more likely to attribute failure to human-like errors rather than inflexibility, leading to reduced perceptions of injustice.

These findings have significant implications for the design of algorithmic management systems in high-stakes areas such as performance management or reward allocation, where perceptions of justice can have a profound effect on employee motivation and engagement. For example, when employees feel that an algorithmic manager is more human-like, they may be more inclined to forgive lapses in performance or errors, which could lead to greater acceptance of these systems in everyday organizational practices [11,25]. Therefore, organizations could reinforce algorithmic management acceptance by considering these psychological aspects when designing and implementing algorithmic management systems, ensuring that they are both effective and perceived as fair by their workforce.

While integrating human-like characteristics into algorithmic management systems can improve their perceived justice and possibly their acceptance, organizations must carefully consider the ethical, technical, and financial aspects of such implementations. *Ethically*, the anthropomorphization of algorithmic managers raises concerns about transparency, accountability, and the potential for deception. Employees must be fully aware that they are interacting with an AI-driven system rather than with a human decision maker to prevent unrealistic expectations regarding affection, discretion, or personalized decision-making. Misrepresenting an algorithm as a human-like entity could create ethical dilemmas, particularly if employees believe that they are engaging with a system capable of understanding context and emotions when, in reality, it follows pre-programmed rules. *Technically*, enhancing AI with anthropomorphic features, such as natural language processing or adaptive decision-making, requires significant advancements, as current systems still struggle with contextual understanding in complex social interactions. *Financially*, developing and maintaining these sophisticated, anthropomorphic AI systems can be costly, particularly for smaller organizations with limited resources. To balance effectiveness and feasibility, organizations can adopt cost-efficient design elements—such as chatbot-like interfaces or personalized feedback mechanisms—that enhance perceived social presence without necessitating highly advanced AI. By addressing these ethical, technical, and financial considerations, organizations can implement algorithmic management systems that are not only effective but also fair and transparent.

### Strengths, limitations, and future directions

This study has several key strengths. First, it builds on different literature from algorithmic management [1], psychological contracts [17], anthropomorphism [20,21], and organizational justice [24] to investigate how employees perceive distributive justice when algorithmic managers fail to fulfill their promises. This approach paves the way for a better understanding of the complex relationship between algorithmic management systems and employee perceptions. Additionally, this research includes two experimental studies that partly support the reliability of the findings. To further enhance the generalizability of the results, Study 2 used alternative vignettes to manipulate promise nonfulfillment and employed more nuanced measures for assessing organizational justice. Finally, this study made an initial attempt to investigate the mediating mechanisms (i.e., perceived rigidity) that help explain the relationship between the nonfulfilled promises of algorithmic managers and distributive justice, offering a more complete and nuanced research design.

Despite its strengths, this study has several limitations. One main limitation of this research is that Study 2 did not replicate either the main effect of the type of nonfulfillment on distributive justice or the direct moderating effect of anthropomorphism on justice that was found in Study 1. Instead, Study 2 revealed a moderated mediation effect: the type of nonfulfilled promises (relational versus transactional) and the degree of anthropomorphism influenced perceptions of distributive justice only indirectly through the perceived rigidity of the algorithmic manager. This suggests that the type of promise nonfulfillment and anthropomorphism may not directly affect justice perceptions but rather shape them by altering how rigid or flexible employees perceive the algorithmic manager to be. Another limitation is that perceived rigidity was assessed via a single-item measure. Although single-item measures can be appropriate for concrete constructs that are easily and directly interpretable [72], this approach inevitably reduces reliability and increases measurement error. As a result, the mediation effect reported in Study 2 should be interpreted cautiously, as it may represent a conservative estimate of the underlying relationship. The absence of additional rigidity-related items in the dataset also prevented internal validation. Future research should therefore employ multi-item scales that capture rigidity in a more reliable and valid manner to more robustly test this mechanism. As such, the observed mediation effect should be interpreted with caution. Additionally, although the experimental manipulations were generally effective across both studies, the effect of the type of nonfulfilled promises on its corresponding manipulation check was only marginally significant (*p* = .06) in Study 1 (yet significant in Study 2). This is a limitation of Study 1, indicating that the distinction between relational and transactional promises may not have been as robustly perceived by participants as intended. Because this manipulation check relied on a single item (and thus reliability may be low) this marginal effect may reflect measurement limitations rather than a complete lack of distinction between the conditions. Moreover, both the mean differences on the manipulation check and the theoretically consistent pattern of effects in the focal analyses suggest that participants nonetheless differentiated the scenarios to some extent. That said, the weak manipulation check limits the certainty with which the Study 1 findings can be interpreted, and we therefore interpret these effects with caution. Furthermore, our anthropomorphism manipulation checks assessed anthropomorphism at a global level and did not include separate items for form anthropomorphism (e.g., human-like names/visuals) versus behavioral anthropomorphism (e.g., conversational/social cues). As a result, we cannot empirically disentangle which facet (form vs. behavioral) drove the observed effects; our findings should therefore be interpreted as reflecting overall anthropomorphism as instantiated by our combined cues. Another limitation is the use of a vignette-based design which, although effective for testing specific hypotheses, restricts the ecological and external validity of the findings. Real-world organizational settings involve more complex dynamics, including factors such as organizational culture, employee–manager relationships, and broader contextual influences (see [73]), all of which may shape perceptions of algorithmic managers’ failure to fulfill promises. Vignettes, admittedly, simplify the complexity of managerial interactions and remove important contextual factors, and it is therefore possible that the effects observed here may manifest differently in actual work environments where employees have ongoing relationships with managers (human or algorithmic), clearer expectations regarding organizational norms, or greater opportunities to contest decisions. This is an important limitation to consider when interpreting the present results. Finally, this study specifically examined the impact of unfulfilled promises by algorithmic managers on distributive justice without testing its effects on the other two key dimensions of organizational justice—procedural and interactional justice [12]. It is possible that unfulfilled promises not only lead employees to perceive unfair outcomes (distributive justice) but also raise concerns about the transparency, consistency, and ethicality of algorithmic decision-making (procedural justice) or the perceived respect and dignity afforded to them by algorithmic systems (interactional justice). However, this study does not provide insights into these aspects.

Future studies could refine the vignettes to strengthen the connection between promises nonfulfillment and the algorithmic manager’s decision-making actions in order to provide clearer insights into how justice perceptions are shaped by the algorithmic manager’s behavior. Specifically, future research should design vignettes that carefully account for potential qualitative differences between the transactional and relational conditions. For example, it should be ensured that neither condition (transactional nor relational nonfulfillment) appears more passive or active based on how the algorithmic manager responds to the promises (e.g., passive neglect versus active rejection of promised actions). Additionally, future research should examine whether the hypothesized relationships are moderated by real-world variables such as employees’ lived experience with algorithmic decision systems (e.g., frequency of use, familiarity, trust), employees’ prior exposure to algorithmic management, and organizational transparency surrounding algorithmic decisions. Incorporating these contextual moderators would substantially enhance the ecological validity of future work and help determine how generalizable the present findings are across different work settings. Furthermore, it would be interesting for future research to investigate how the effects of anthropomorphism on algorithmic managers evolve over time. While this study demonstrates the impact of human-like traits on justice perceptions in the short term, long-term effects on employee attitudes toward or acceptance of these systems remain largely underexplored. Additionally, future research should investigate in a more systematic manner employees’ reactions to algorithmic management’s behaviors in terms of procedural and interactional justice [12], as these facets may provide additional insights into how employees perceive and respond to decision-making processes. Specifically, exploring how factors such as transparency, consistency, and the quality of communication from algorithmic managers can influence justice perceptions could help in understanding the broader context of organizational justice in algorithm-driven environments. In a similar vein, future research could introduce procedural factors such as explanations or opportunities for recourse to examine whether providing procedural fairness elements reduces or overrides the influence of anthropomorphism on distributive justice perceptions. Furthermore, it would be interesting if future research examined whether increasing the sophistication of anthropomorphic features—such as advanced emotional recognition, adaptive learning, or deeper conversational capabilities—could enhance the perceived ability of algorithmic managers to uphold relational commitments. Accordingly, researchers could explore whether the anthropomorphism of algorithmic managers could influence employees’ justice-related reactions to relational (besides transactional) promise nonfulfillment. Additionally, since employee familiarity with technology and digital tools may shape employees’ perceptions of algorithmic managers’ justice, future research should explore how professional sectors (e.g., the IT sector) influence employees’ perceptions of justice when promises are unfulfilled. Besides, building on the current findings, future studies could extend our design by incorporating the *valence* of the algorithmic manager’s behavior (positive vs. negative outcomes) alongside promise type and anthropomorphism (e.g., a 2 × 2 × 2 design) to directly test potential negativity biases in fairness judgments. Crucially, although we conceptualized perceived rigidity as a mediating variable in the relationship between promise nonfulfillment and distributive justice, alternative causal pathways cannot be ruled out. More specifically, justice perceptions may influence how rigid an algorithmic manager is perceived to be, meaning rigidity could function as an outcome rather than a mediator. To explore this possibility, we conducted an exploratory reverse mediation analysis in Study 2 in which distributive justice served as the mediator and rigidity as the outcome. This reverse pathway was not significant, providing some preliminary, though not sufficient, support for the proposed causal direction. Nevertheless, because our vignette design does not temporally separate these constructs, we cannot definitively establish causal direction. Thus, the mediation effect in Study 2 should be interpreted as one theoretically coherent pathway among several possible alternatives. Future research employing longitudinal or multi-wave designs is needed to test the directionality of these relationships more rigorously.

Finally, while our findings suggest that anthropomorphization can enhance employees’ perceptions of fairness in algorithmic management, this raises important ethical questions about whether such design choices genuinely improve organizational justice or merely create the illusion of fairness. Relying on anthropomorphization alone may risk diverting attention from more fundamental concerns, such as transparency, accountability, and employees’ ability to contest algorithmic decisions. If anthropomorphization is used as a mechanism to ‘soften’ the perception of rigid or unfair algorithmic decisions, organizations may inadvertently suppress employees’ critical awareness of systemic biases embedded in algorithmic decision-making. Thus, future research and practice should move beyond surface-level design features and explore ways to embed substantive fairness principles—such as responsible and ethical leadership—into algorithmic management systems.

## Conclusions

Taken together, the findings of this study provide partial evidence that employees may perceive non-anthropomorphized algorithmic managers as more rigid when transactional (as opposed to relational) promises are unfulfilled, which in turn can lower perceived distributive justice. Therefore, anthropomorphic (i.e., human-like) qualities in algorithmic management systems may buffer negative reactions to such unfulfilled promises by reducing perceptions of rigidity. However, the divergent pattern across the two studies (particularly the absence of the direct interaction effect in Study 2) suggests that these effects are not uniformly robust. Instead, the findings may suggest conditional support for the proposed model: the interaction between anthropomorphism and type of promise nonfulfillment may depend on how employees construe or evaluate distributive justice (self-referential vs. generalized approach) and on the salience of the relational–transactional distinction. Accordingly, the conclusions should be interpreted with caution, as the mixed results point to important boundary conditions that warrant further theoretical refinement and empirical investigation. Practically, the study suggests that organizations could reinforce algorithmic management acceptance by integrating human-like characteristics into algorithmic management systems. However, there are ethical concerns about prompting employees to perceive algorithmic managers as human, as excessive anthropomorphization could obscure boundaries and weaken accountability.

## Supporting information

S1 File S1 inclusivity questionnaire. (DOCX)

S2 File S2 online supplemental material. (DOCX)

S3 File S3 test of assumptions study 1. (DOCX)

S4 File S4 sensitivity analyses for manipulations. (DOCX)

S5 File This is the S5 test of assumptions study 2. (DOCX)
