# Supplementary material for: When algorithmic managers fail to fulfill their promises: The role of anthropomorphism in shaping justice perceptions
Source: PLoS One. 2026 Feb 20;21(2):e0340860. doi: 10.1371/journal.pone.0340860 (PMC12923041; doi:10.1371/journal.pone.0340860)
Supplement: S2 File — (DOCX) [file pone.0340860.s002.docx]

**Online Supplemental Material**

**Studies 1 and 2**

**Attitudes towards AI Scale**

**Below you will find sentences about your general attitude toward Artificial Intelligence (AI).**
 Please indicate to what extent you agree with the following statements:

I believe that AI will improve my life

I believe that AI will improve my work.

I think I will use AI technology in the future.

I think AI technology is positive for humanity.

(1 = *Not at all*; 10 = *Completely Agree*)

**Low Anthropomorphism Condition**

Beta Management Company is launching a new range of robot managers. Imagine that you are an employee at Beta Management Company where a robot manager is your manager! Beta Management Company introduces your newly assigned robot manager, Robo3000:

**“*Robo3000 will be your manager, providing instructions on various tasks. Robo3000 is stationed in the IT department but can be contacted via a chat tool for any issues.***

***Robo3000 will guide you on various tasks. You can notify Robo3000 when tasks are completed. Robo3000 will request updates on your progress throughout the day.***

***Robo3000 operates 24/7, without breaks, except for one day of maintenance each month*.”** Here is a picture of your **Robo3000**:

**Figure S1.** Low-anthropomorphic algorithmic manager.

**
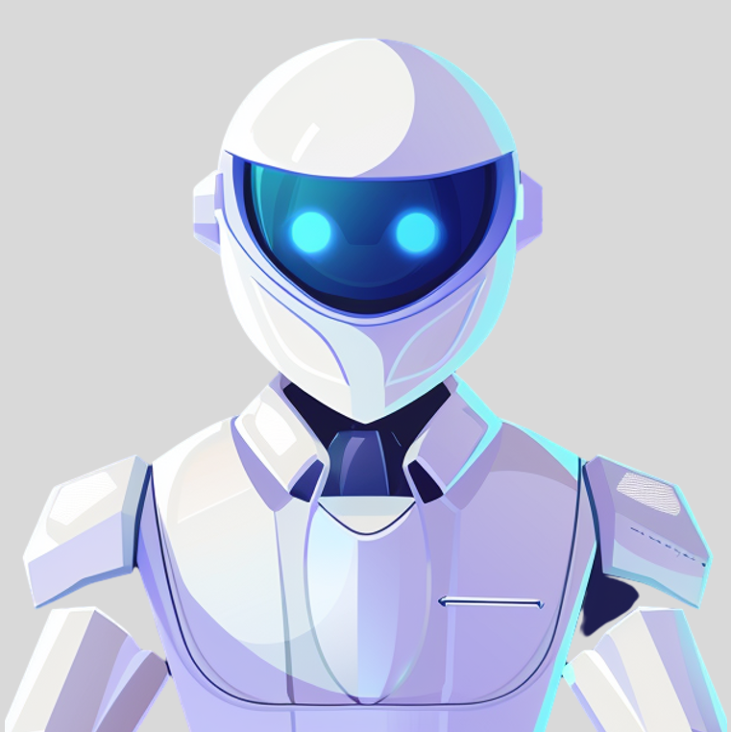
**

*Note.* AI-generated stimulus image created using Midjourney and DALL·E 3 (prompt-based generation). Prompts were designed to depict an explicitly non-human, robot-like algorithmic manager, minimizing human facial features and social cues, while maintaining neutrality in terms of visual salience and professionalism. This figure served as the low-anthropomorphism comparison condition in the experimental manipulation.

After introducing Robo3000, Beta Management Company, responsible for its production and maintenance, invites you to consider the specifications and work-related functions of Robo3000.

 Please write down two ideas that come to mind about how Robo3000 functions at work, considering that it is a robot rather than a real person.

________________________________________________________________

________________________________________________________________

________________________________________________________________

________________________________________________________________

________________________________________________________________

**High Anthropomorphism Condition – Female Version**

Beta Management Company is launching a new range of robot managers. Imagine that you are an employee at Beta Management Company where a robot manager is your manager!     The robot manager introduces herself to you with the name Lily Adams and tells you: **“*Hello, I am Lily Adams, your new manager and companion at work. My office is on the same floor as yours, so feel free to visit me if you need anything. We will work closely on various tasks. After assigning tasks, I will monitor your progress. You can ask me directly for clarifications or help whenever needed. When you complete a task, contact me directly so we can discuss your deliverables and plan the next steps. I am available during normal office hours, just like everyone else in the company*.”** Here is a picture of your robot manager **Lily Adams:**

**Figure S2.** High-anthropomorphism algorithmic manager (female version).
**
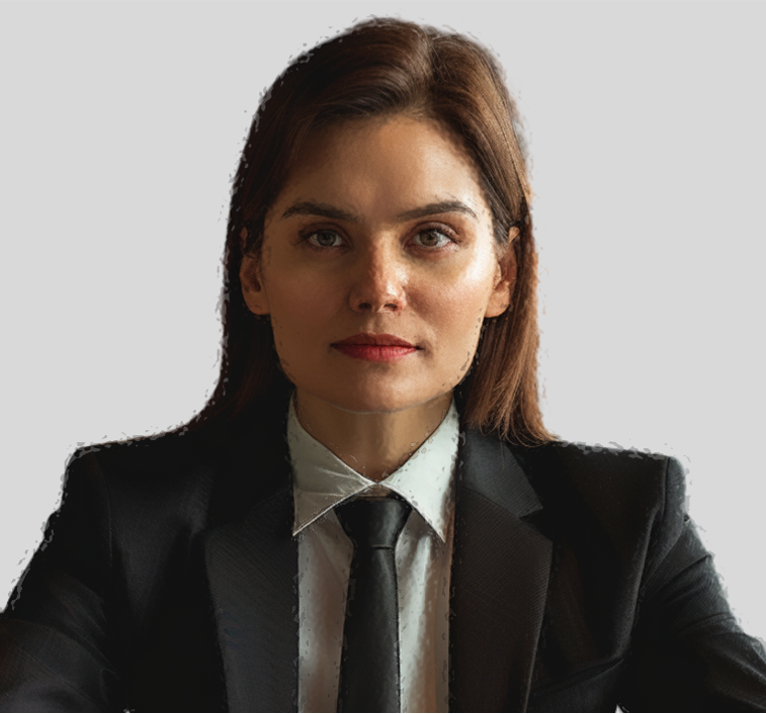
**

*Note.* AI-generated stimulus image created using Midjourney and DALL·E 3 (prompt-based generation). Prompts were constructed to keep visual characteristics neutral and comparable (e.g., attractiveness, age, perceived competence) while manipulating human likeness (anthropomorphism level).

After introducing herself, Lily Adams invites you to consider what kind of person she/he is when interacting with others at work.

 Please try to think of her as a person rather than a robot and write down two ideas that come to mind about the type of person you think Lily Adams is at work.

________________________________________________________________

________________________________________________________________

________________________________________________________________

________________________________________________________________

**High Anthropomorphism Condition – Male Version**

Beta Management Company is launching a new range of robot managers. Imagine that you are an employee at Beta Management Company where a robot manager is your manager!     The robot manager introduces himself to you with the name Noah Adams and tells you: **“*Hello, I am Noah Adams, your new manager and companion at work. My office is on the same floor as yours, so feel free to visit me if you need anything. We will work closely on various tasks. After assigning tasks, I will monitor your progress. You can ask me directly for clarifications or help whenever needed. When you complete a task, contact me directly so we can discuss your deliverables and plan the next steps. I am available during normal office hours, just like everyone else in the company*.”** Here is a picture of your robot manager **Noah Adams:**

**Figure S3.** High-anthropomorphism algorithmic manager (male version).

**
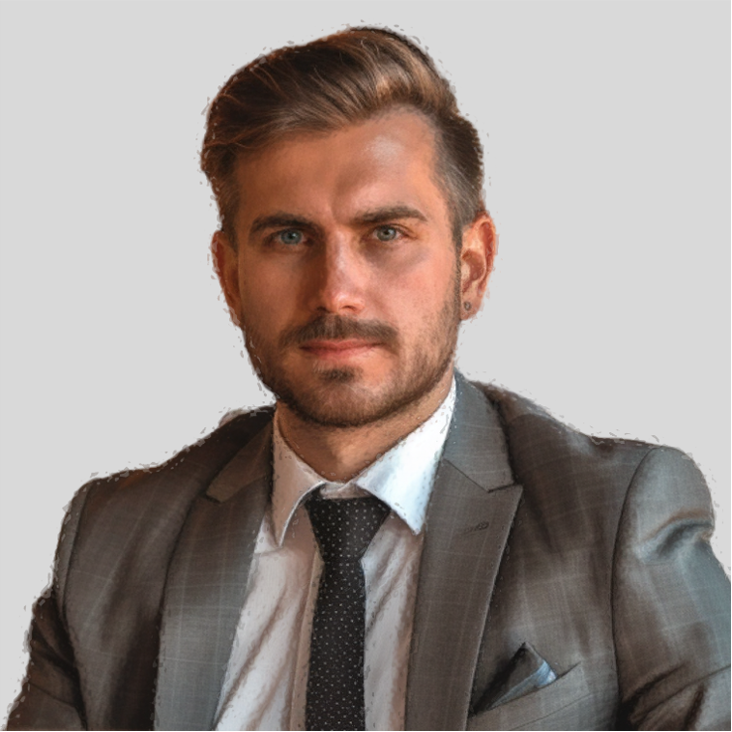
**

*Note.* AI-generated stimulus image created using Midjourney and DALL·E 3 (prompt-based generation). Prompts were constructed to keep visual characteristics neutral and comparable (e.g., attractiveness, age, perceived competence) while manipulating human likeness (anthropomorphism level).

After introducing himself, Noah Adams invites you to consider what kind of person she/he is when interacting with others at work.

 Please try to think of him as a person rather than a robot and write down two ideas that come to mind about the type of person you think Noah Adams is at work.

________________________________________________________________

________________________________________________________________

________________________________________________________________

**Study 1**

**Nonfulfillment of Transactional Promises Condition:**

"During your contract negotiation meetings with your robot manager, it becomes clear that your Robot Manager is committed to ensuring that you complete your assigned tasks and achieve company goals. Following discussions with your Robot Manager, you can anticipate:
**Consistent Salary Benchmarking**:
 Your robot manager assured you that your salary would be regularly benchmarked against industry standards and comparable companies. This ensures that your compensation is aligned with market trends.
**Annual Salary Raises for Standard of Living:**
 Based on discussions with your robot manager, you can expect annual salary raises to keep pace with inflation and maintain your standard of living. These adjustments are typically scheduled on a yearly basis and are subject to company performance and economic conditions.
**Regular Bonuses Every 6 Months**:
 Your robot manager outlined a structured bonus policy where you will receive bonuses every six months, contingent upon achieving predefined performance targets. These bonuses serve as incentives to reward your contributions to the organization’s success and are part of the company’s commitment to recognizing and motivating high performance.

 Your Robot Manager’s promises are designed to provide tangible benefits tied to your performance and market conditions. By focusing on competitive compensation and performance-based rewards, your robot manager aims to align your expectations with measurable outcomes and company goals."

-----------------------------------------------TWO YEARS LATER--------------------------------------

Now, imagine that you have been working for this organization for two full years after these contract arrangements have been made. You have demonstrated remarkable commitment and exerted tremendous effort throughout this time.


 However...

 -For these two years, your robot manager has not given regular bonuses.
 -Moreover, your salary has never been benchmarked according to the increase in the cost of living.
 -Recently, your performance evaluation was exceptional, but your robot manager did not give you the pay raise you were promised.

**Nonfulfillment of Relational Promises Condition:**

"During your contract negotiation meetings with your robot manager, it becomes clear that your Robot Manager is committed to ensuring that you achieve your professional goals but also feel supported and valued in your personal life. Following discussions with your Robot Manager, you can anticipate:

**Specialised Training Workshops:**  Your Robot Manager promises to provide you with access to ongoing specialised training workshops designed to enhance your skills and make you more competitive in the external job market. These workshops are tailored to your career aspirations and are aimed at helping you achieve long-term growth.

**Opportunities to Expand Your Professional Network:** Your Robot Manager ensures that you will have ample opportunities to attend industry conferences, seminars, and networking events. These events are aimed at helping you build valuable connections with industry leaders and peers, fostering a strong professional network that can support your career development.

**Support with Personal or Family-Related Issues:** Your Robot Manager is dedicated to supporting your overall well-being. This includes assistance with personal or family-related issues, such as flexible work arrangements, access to counselling services, and family support programs. Your Robot Manager’s goal is to ensure that you can maintain a healthy work-life balance and feel fully supported in both your personal and professional life.    Your Robot Manager’s promises are designed to create a supportive and enriching work environment, fostering long-term loyalty and satisfaction. By focusing on your overall well-being and career development, your Robot Manager aims to build a strong, trust-based relationship with you, ensuring that you feel valued and motivated to contribute your best to the company."

-----------------------------------------------TWO YEARS LATER--------------------------------------

 Now, imagine that you have been working for this organization for two full years after these contract arrangements have been made. You have demonstrated remarkable commitment and exerted tremendous effort throughout this time.

 However...
 -A year ago, you applied for a training workshop held in the company that would help you develop new skills. However, your robot manager did not approve it.
 -Moreover, you have never been given the opportunity to expand your professional network within and outside the organization.
 -Recently, you were dealing with some important personal issues, so you submitted a request for time off to your robot manager, but again, your robot manager did not approve your request.

**Manipulation Check Item for Type of Promises (relational/ transactional)**

Based on the scenario that you read...

Would you characterize the nature of the promises made by your Robot Manager:

1 = *Relational* (eg. promoting mutual respect and engaging interactions), 7 = *Transactional* (e.g. promoting instrumental relations and focusing on task completion

**Manipulation Check Items for Perceived Anthropomorphizing of the Algorithmic Manager**

Did the robot manager’s appearance lead you to anthropomorphize it (=perceive it as more human-like) by attributing human-like thoughts, feelings, or intentions to him/her?

How interactive and engaging does your collaboration with your robot manager feel?

(1 = *Not at all*, 7 = *To a great extent*)

**Measures Study 1**

**Perceived Distributive Justice (Colquitt, 2011)**

If what you read in the above scenario had happened in real life, to what extent would you feel that…

The outcomes you received from your robot manager reflect the effort you have put into your work.

The outcomes you received from your robot manager were appropriate for the work you have completed.

The outcomes you received from your robot manager reflect what you have contributed to your work.

The outcomes you received from your robot manager are justified, given your performance.

(1 = *To a very small extent*; *To a very large extent*)

**Study 2**

**Nonfulfillment of Transactional Promises Condition:**

During your project planning discussions with your robot manager, it becomes clear that your robot manager is focused on ensuring your productivity aligns directly with company objectives. Following these discussions, you can expect:

**Defined Task Milestones with Timely Bonuses:**

Your robot manager sets clear deadlines for task completion and offers a structured bonus system, where bonuses are paid upon reaching specific milestones. These bonuses are tied strictly to the timely and accurate completion of assigned tasks.

**Fixed Work Schedule with Overtime Compensation:**

Your robot manager enforces a work schedule with clear start and end times. If overtime is required, it will be compensated at a set rate. Your earnings will increase proportionally based on the hours worked beyond your regular schedule.

**Annual Performance Review for Merit Increases:**

Your robot manager conducts an annual review of your performance, assessing your output and contributions. Merit-based salary increases are offered depending on whether you meet or exceed the targets that have been established at the start of the year.

Your robot manager’s promises are focused on efficiency, measurable outputs, and providing direct financial rewards for your performance. This transactional approach ensures that

your efforts are tied directly to tangible outcomes, creating a clear and transparent exchange between your work and compensation.

-----------------------------------------------TWO YEARS LATER--------------------------------------

Now, imagine that you have been working for this organization for two full years after these project arrangements were made. You have demonstrated remarkable commitment and exerted tremendous effort throughout this time.

However...

-Your robot manager promised milestone-based bonuses but over these two years, you have

not received them.

-Additionally, your salary was supposed to be adjusted to reflect your overtime work, but no adjustments have been made.

-Recently, despite achieving all your task milestones, your robot manager did not grant the merit-based salary increase you were promised.

**Nonfulfillment of Relational Promises Condition:**

During your project planning discussions with your robot manager, it becomes clear that your robot manager is focused on nurturing your overall professional development and personal satisfaction. Following these discussions, you can expect:

**Custom Mentorship Program:**

Your robot manager organizes a one-on-one mentorship program with an experienced leader in your field, tailored to your unique career goals. This program focuses not just on skill acquisition but also on navigating workplace challenges and long-term career development strategies.

**Personal Growth Opportunities:**

Your robot manager encourages you to participate in workshops on topics of personal interest, such as creative thinking and communication skills. These opportunities are designed to enhance your well-being and help you develop both personally and professionally.

**Holistic Wellness Initiatives**

Your robot manager prioritizes your mental and physical health by offering wellness initiatives that include meditation classes, gym memberships, and mental health trainings. These initiatives are aimed at ensuring you feel supported and can maintain a healthy work-life balance.

Your robot manager’s promises focus on your broader life satisfaction, helping you to grow not only in your current role but also as an individual. This relational approach is designed to build t

rust and commitment, leading to long-term loyalty and engagement

-----------------------------------------------TWO YEARS LATER--------------------------------------

Now, imagine that you have been working for this organization for two full years after these project arrangements were made. You have demonstrated remarkable commitment and exerted tremendous effort throughout this time.

However...

-A year ago, you were supposed to start a personalized mentorship program that would help you with your career goals, but your robot manager did not organize it as promised.

-You were also expecting to participate in workshops on personal growth, but no such opportunities were made available to you.

-Recently, you submitted a request for time off to manage personal well-being, but your robot manager did not approve it.

**Measures Study 2**

**Perceived Distributive Justice**

I believe that the rewards and recognition employees would receive from such a robot manager would be fair and based on employee performance.

I believe that such a robot manager would allocate tasks and responsibilities equitably (fairly) among team members.

The benefits employees would gain from such a robot manager would be proportional to their contributions to the team.

Such a robot manager would acknowledge and compensate for employee efforts appropriately.

(1 = *To a very small extent*, 7 = *To a very large extent*)
