# Supplementary material for: When algorithmic managers fail to fulfill their promises: The role of anthropomorphism in shaping justice perceptions
Source: PLoS One. 2026 Feb 20;21(2):e0340860. doi: 10.1371/journal.pone.0340860 (PMC12923041; doi:10.1371/journal.pone.0340860)
Supplement: S3 File — (DOCX) [file pone.0340860.s003.docx]

**Supporting Material – Study 1**

**Test of Assumptions for Regression Analysis – Study 1**

**Normality**

Normality was tested by means of a Q-Q plot and the Shapiro-Wilk test. The plot shows that the residuals are largely normally distributed (Fig 1).

**Fig 1. Normal Q-Q Plot (Study 1)**


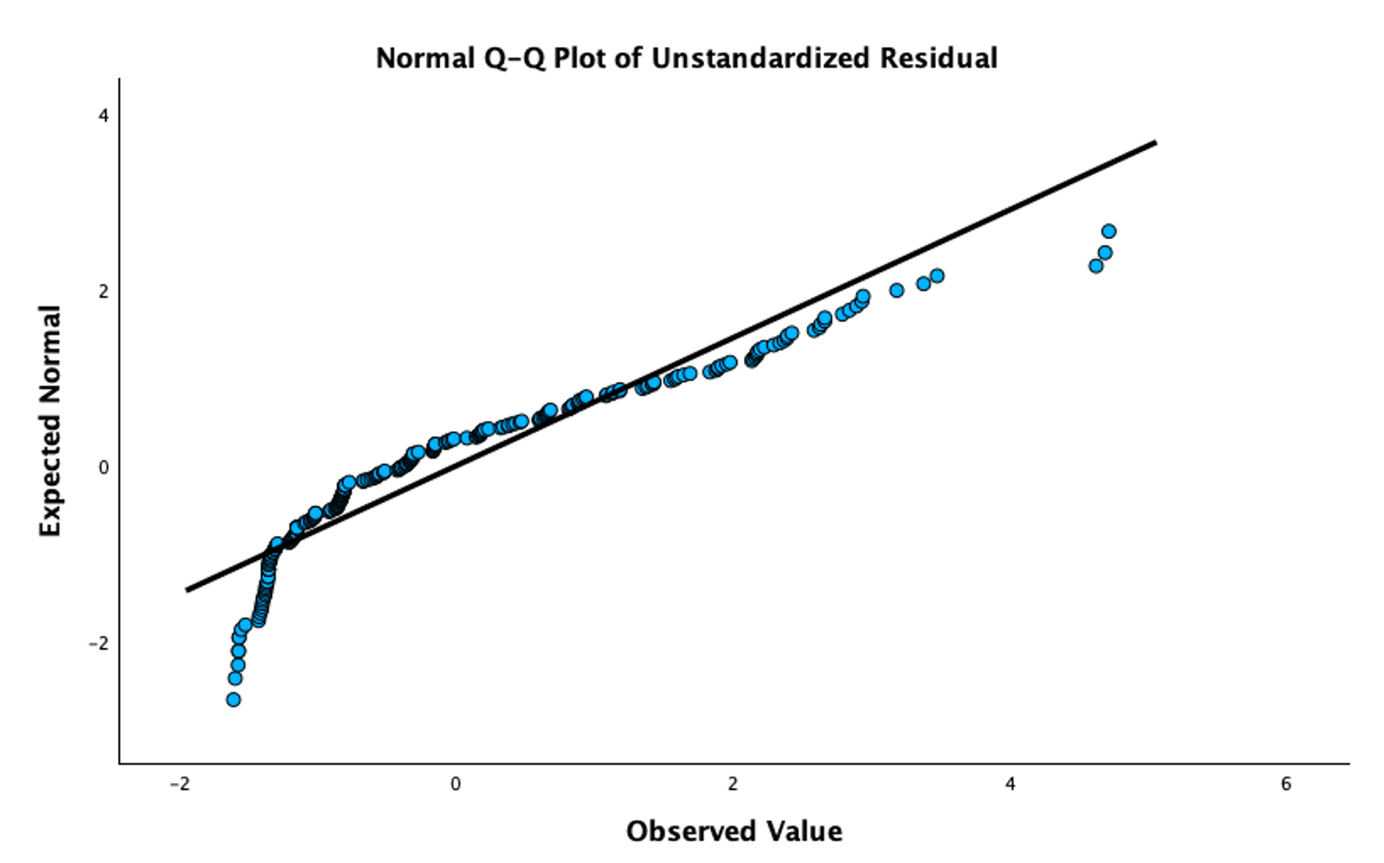


**Multicollinearity**

The VIF values of variables in Study 1 range between 1.01 (tenured position) and 2.01 (Nonfulfillment). Because these VIF values are below or close to 2, they document the absence of multicollinearity.

**Homoscedasticity**

Homoscedasticity was tested by plotting the predicted values (DV=distributive justice) against residuals (Fig 2). The plot shows a rectangular distribution of values and the assumptions for regression are met. In addition, we ran the robust Breusch-Pagan test because the residuals are not perfectly normally distributed. The test was not statistically significant (p=.078) but close to statistical significance.

**Fig 2. Test of Homoscedasticity (Study 1)**


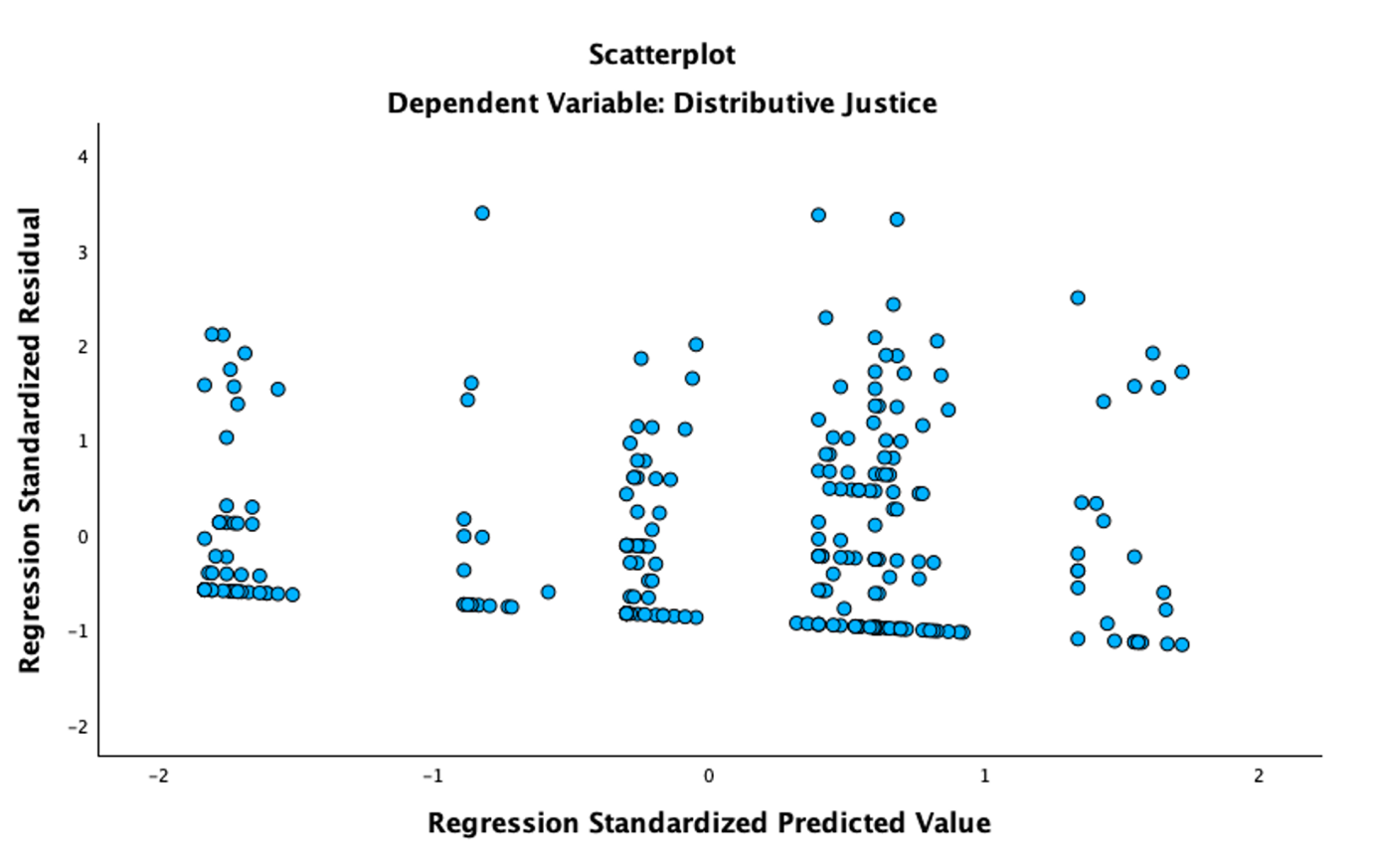


Because the assumption of homoscedasticity was not perfectly met, we reran our analysis with robust standard errors (HC3) to justify the interpretation of our analysis in PROCESS. The results of our model with HC3 are shown in Table 1. Although the standard errors are higher compared to PROCESS, the same relationships were significant. We thus continue to interpret the model output from PROCESS.

**Table 1. Model Study 1 with Parameter Estimates with Robust Standard Errors (HC3)**

|  | *B* | *Robust SE* | *p* | *CI* |
| --- | --- | --- | --- | --- |
| Constant | 2.83 | .60 | .00 | 1.64,4.01 |
| AI attitude | -.01 | .06 | .84 | -.13,.10 |
| Tenured position | -.21 | .24 | .36 | -.68,.25 |
| Nonfulfillment | -.51 | .24 | .04 | -.98,-.03 |
| Anthropomorphism | -.16 | .23 | .49 | -.61,.29 |
| Nonfulfillment* anthropomorphism | .71 | .35 | .04 | .02,1.41 |
| R^2^ | .03 |  | .24 |  |

**Influence Diagnostics – Study 1**

Studentized residuals range between -1.18 and 3.44 and do not indicate a particular influence of single cases.

The values of leverage range between -1.18 and 3.44. Based on the number of predictors and the sample size, a threshold of .05 indicates a particular influence of single cases. However, many of the cases in our study show an influence above the defined threshold.

Cook’s Distance ranges between .00 and .057. Based on the sample size, a threshold of .05 indicates a particular influence of single cases. Only one case lies above this threshold.

Based on the sample size, a threshold of .125 indicates a particularly high/low DFBETA and a related influence of this case. PROCESS identifies one case with a DFBETA higher than .125 in the constant. All other DFBETAs lie below this threshold.

Because the diagnostics do not report a consistent influence of single cases, we decided to retain all cases for our analysis.

**Moderation – Study 1**

**Johnson-Neyman Plot**

The Johnson-Neyman plot (Fig 3) for the interaction between anthropomorphism and nonfulfillment illustrates that the interaction is significant for high values of nonfulfillment only (i.e., transactional non-fulfillment). The plot was produced with the package interactions in R (Long, 2024).

**Fig 3. Johnson-Neyman Plot**

**
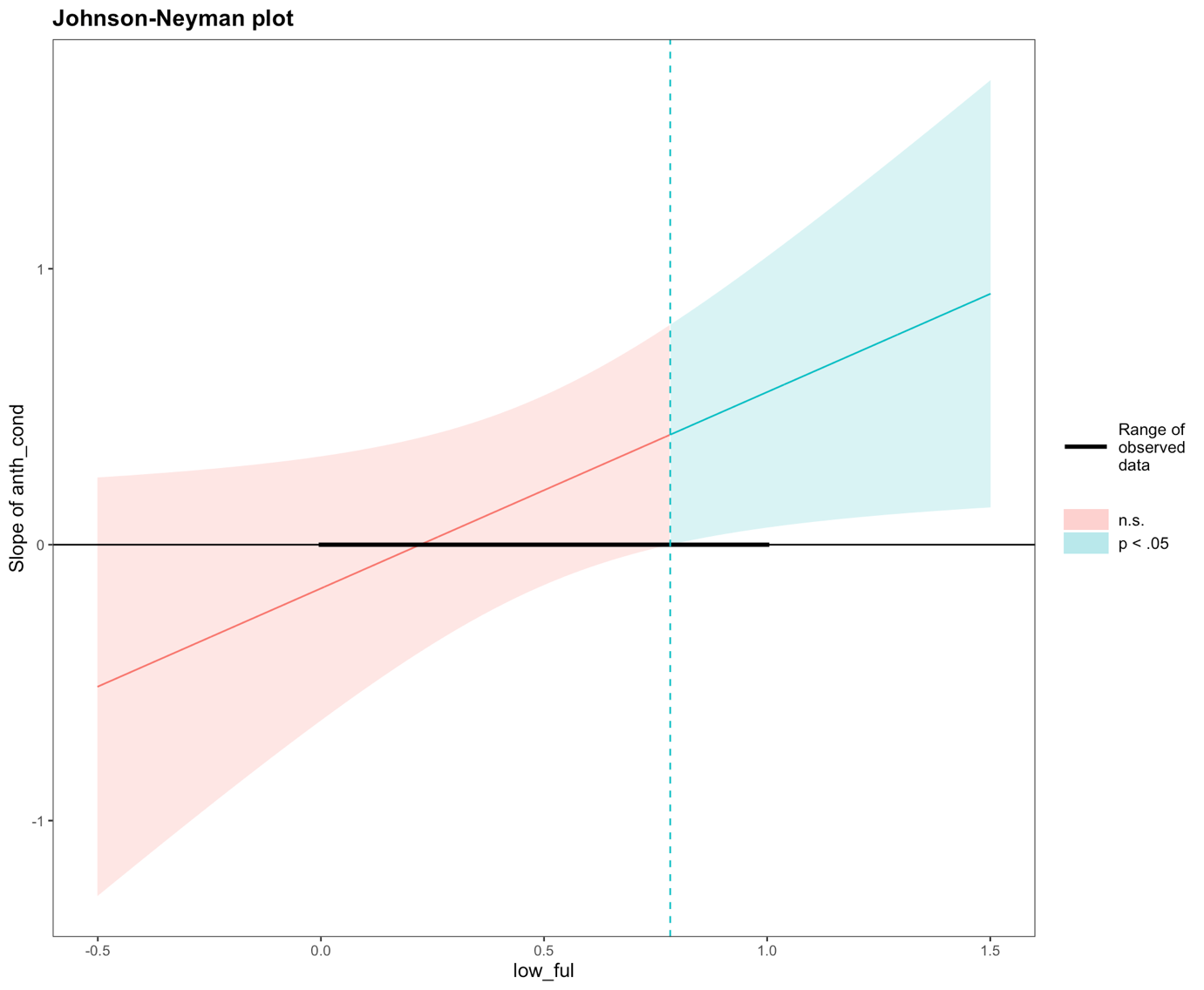
**

*Note.* Low_ful = nonfulfillment (0=relational, 1=transactional) ;anth_cond = anthropomorphism (0=low, 1=high)

**Reference**

Long J. A. (2024). interactions: comprehensive, user-friendly toolkit for probing interactions. R package version 1.2.0. [https://CRAN.R-project.org/package=interactions](https://cran.r-project.org/package=interactions)
