# Supplementary material for: When algorithmic managers fail to fulfill their promises: The role of anthropomorphism in shaping justice perceptions
Source: PLoS One. 2026 Feb 20;21(2):e0340860. doi: 10.1371/journal.pone.0340860 (PMC12923041; doi:10.1371/journal.pone.0340860)
Supplement: S4 File — (DOCX) [file pone.0340860.s004.docx]

Table S4.1

Sensitivity analysis with bias-corrected and accelerated bootstrap (Study 1) predicting distributive justice

|  | *B*(*SE*) | *Bias* | *CI (95%)* |
| --- | --- | --- | --- |
| Constant | 2.83(.54)*** | .000 | 1.661, 4.011 |
| AI attitude | -.01(.06) | -.001 | -.131, .102 |
| Tenured position | -.21(.21) | .001 | -.668, .229 |
| Nonfulfillment | -.51(.25)* | .004 | -.972, -.008 |
| Anthropomorphism | -.16(.24) | -.000 | -.608, .284 |
| Nonfulfillment* anthropomorphism | .71(.35)* | -.003 | .039, 1.392 |
| R^2^ | .03 |  |  |

*Note.* Bootstrap results are based on 5000 bootstrap samples.

Table S4.2

Sensitivity analysis with bias-corrected and accelerated bootstrap predicting distributive justice (Study 2)

|  | *B*(*SE*) | *Bias* | *CI (95%)* |
| --- | --- | --- | --- |
| Constant | 2.44(.75)** | -.010 | .907, 3.930 |
| AI attitude | .23(.06)*** | .003 | .111, .342 |
| Tenured position | .17(.25) | .000 | -.308, 651 |
| Nonfulfillment | -.02(.29) | -.023 | -.591, .554 |
| Anthropomorphism | .35 (.29) | -.013 | -.192, .881 |
| Nonfulfillment* anthropomorphism | -.21(.40) | .012 | -.981, .599 |
| Rigidity | -.20(.07)** | .000 | -.349, -.045 |
| R^2^ | .10*** |  |  |

*Note.* Bootstrap results are based on 5000 bootstrap samples.

Table S4.3

Sensitivity analysis with bias-corrected and accelerated bootstrap predicting rigidity (Study 2)

|  | *B*(*SE*) | *Bias* | *CI (95%)* |
| --- | --- | --- | --- |
| Constant | 5.32(.63)*** | -.026 | 3.927, 6.545 |
| AI attitude | .00(.05) | .003 | -.099, .117 |
| Tenured position | .00(.24) | .004 | -.455, .483 |
| Nonfulfillment | .77(.27)** | -.005 | .185, 1.374 |
| Anthropomorphism | .64(.27)* | .000 | .083, 1.227 |
| Nonfulfillment* anthropomorphism | -.89(.38)* | .000 | -1.628, -.172 |
| R^2^ | .04 |  |  |

*Note.* Bootstrap results are based on 5000 bootstrap samples.
