# Supplementary material for: When algorithmic managers fail to fulfill their promises: The role of anthropomorphism in shaping justice perceptions
Source: PLoS One. 2026 Feb 20;21(2):e0340860. doi: 10.1371/journal.pone.0340860 (PMC12923041; doi:10.1371/journal.pone.0340860)
Supplement: S5 File — (DOCX) [file pone.0340860.s005.docx]

**Supporting Material – Study 2**

**Test of Assumptions for Regression Analysis – Study 2**

**Normality**

Normality was tested by Q-Q plots and the Shapiro-Wilk test. The plots show that the residuals are largely normally distributed (Fig 1 and 2).

**Fig 1. Normal Q-Q Plot, dependent variable distributive justice (Study 2)**


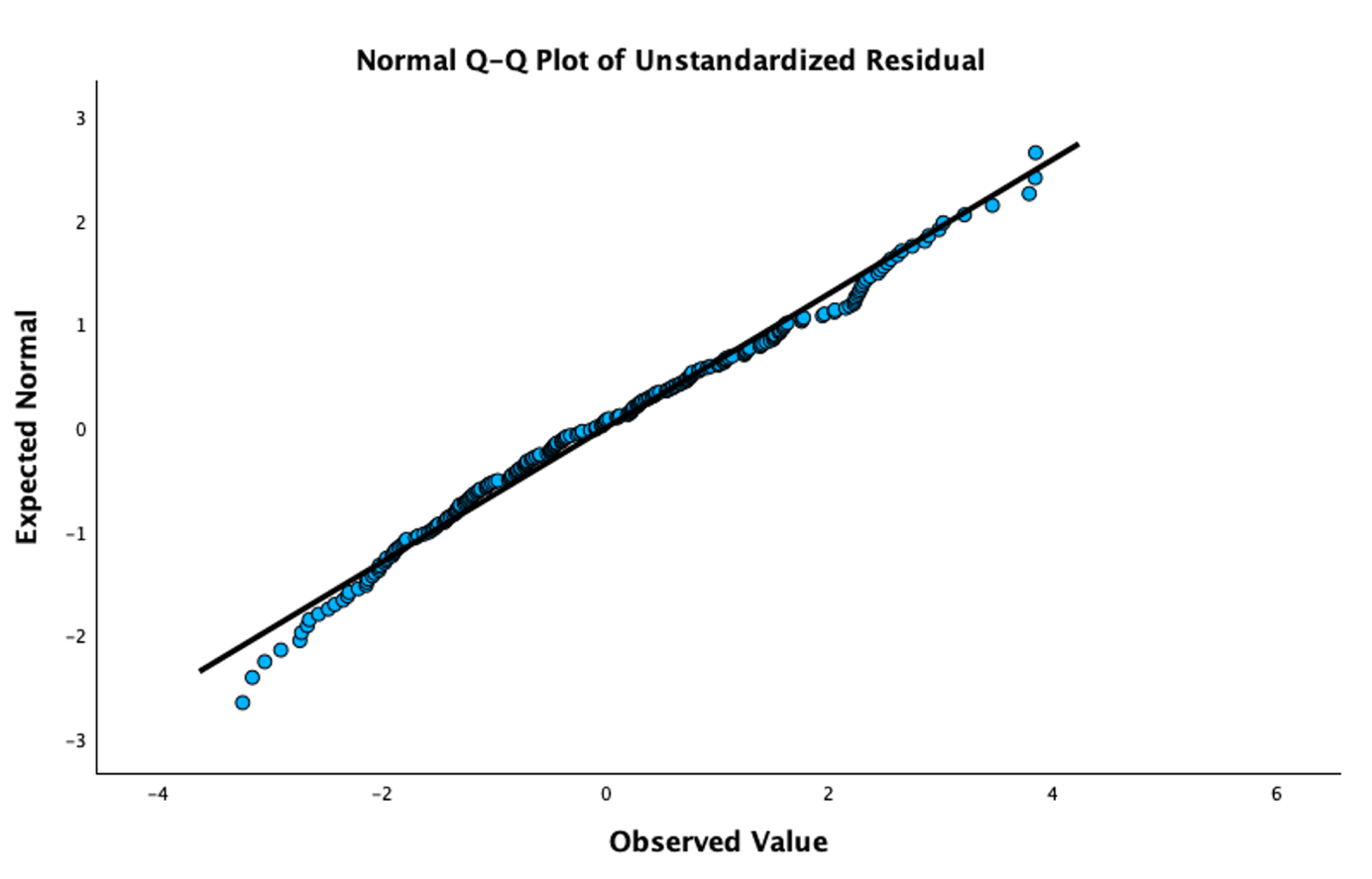


**Fig 2. Normal Q-Q Plot, dependent variable rigidity (Study 2)**

**
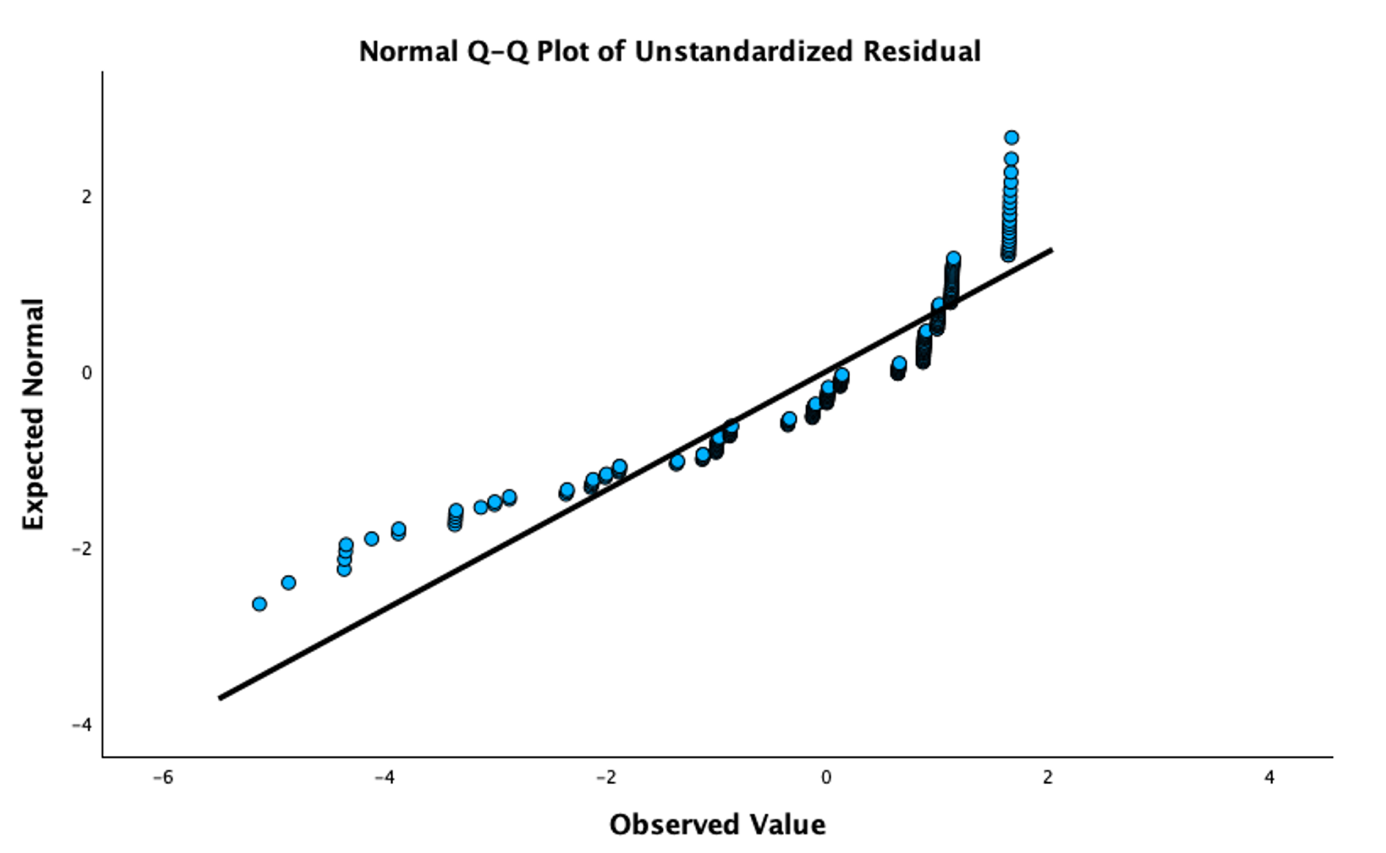
**

**Multicollinearity**

When estimating the mediator rigidity, the VIF values of variables in Study 2 range between 1.01 (AI attitude) and 2.07 (anthropomorphism). When estimating the dependent variable distributive justice, the VIF values range between 1.01 (tenured position) and 2.16 (anthropomorphism). Because these VIF values are below or close to 2, they document the absence of multicollinearity.

**Homoscedasticity**

Homoscedasticity was tested by plotting the predicted values (DV=distributive justice/rigidity) against residuals (Fig 3 and 4). The plot for perceived rigidity shows a rectangular distribution of values and the assumptions for regression are met. The plot for distributive justice fails to present a rectangular distribution and the assumptions for regression are not perfectly met. In addition, we ran the robust Breusch-Pagan test because the residuals are not perfectly normally distributed. The tests for both models were statistically significant justice (p=.022/.001).

**Fig 3. Test of Homoscedasticity, dependent variable distributive justice (Study 2)**


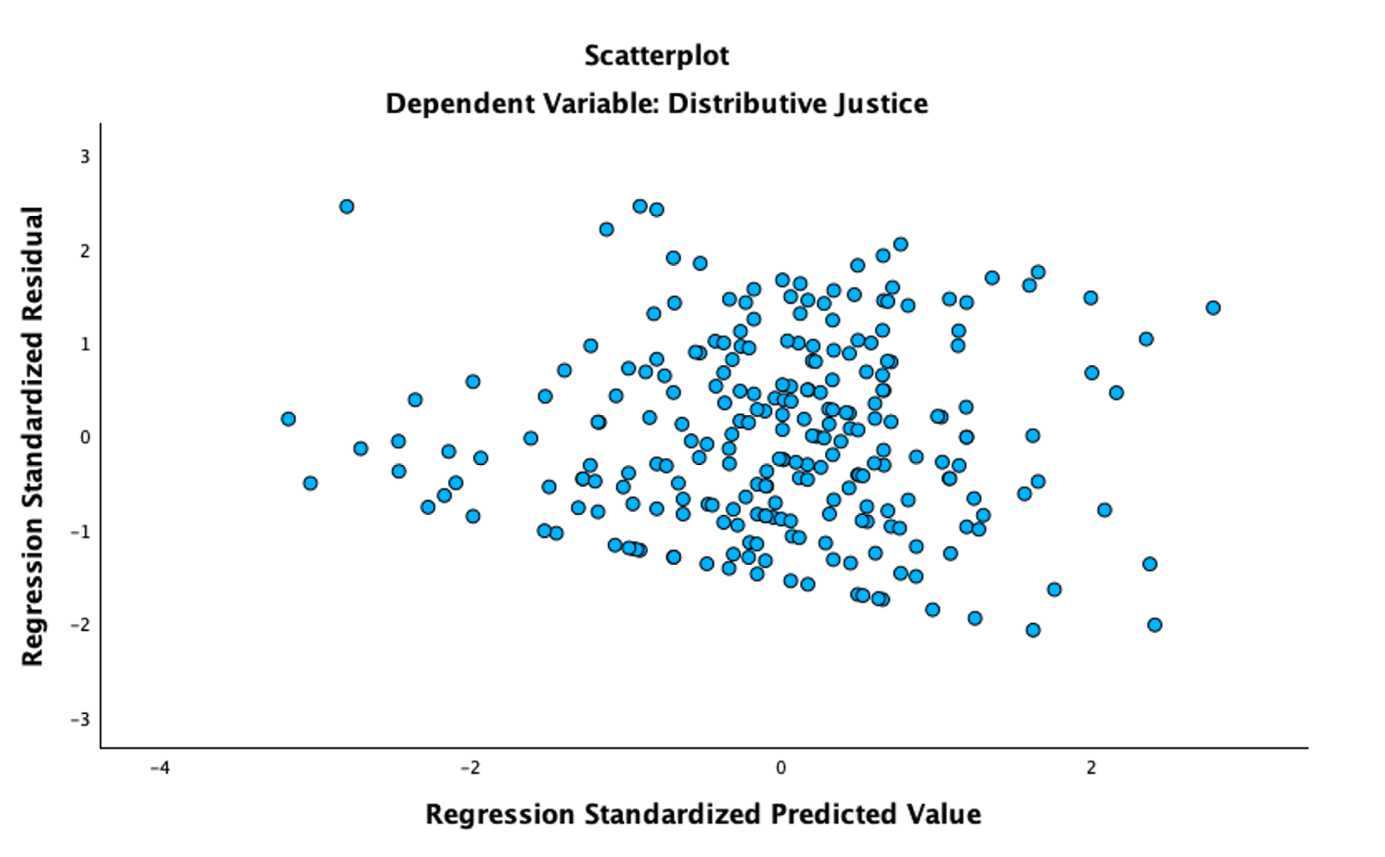


**Fig 4. Test of Homoscedasticity, dependent variable rigidity (Study 2)**


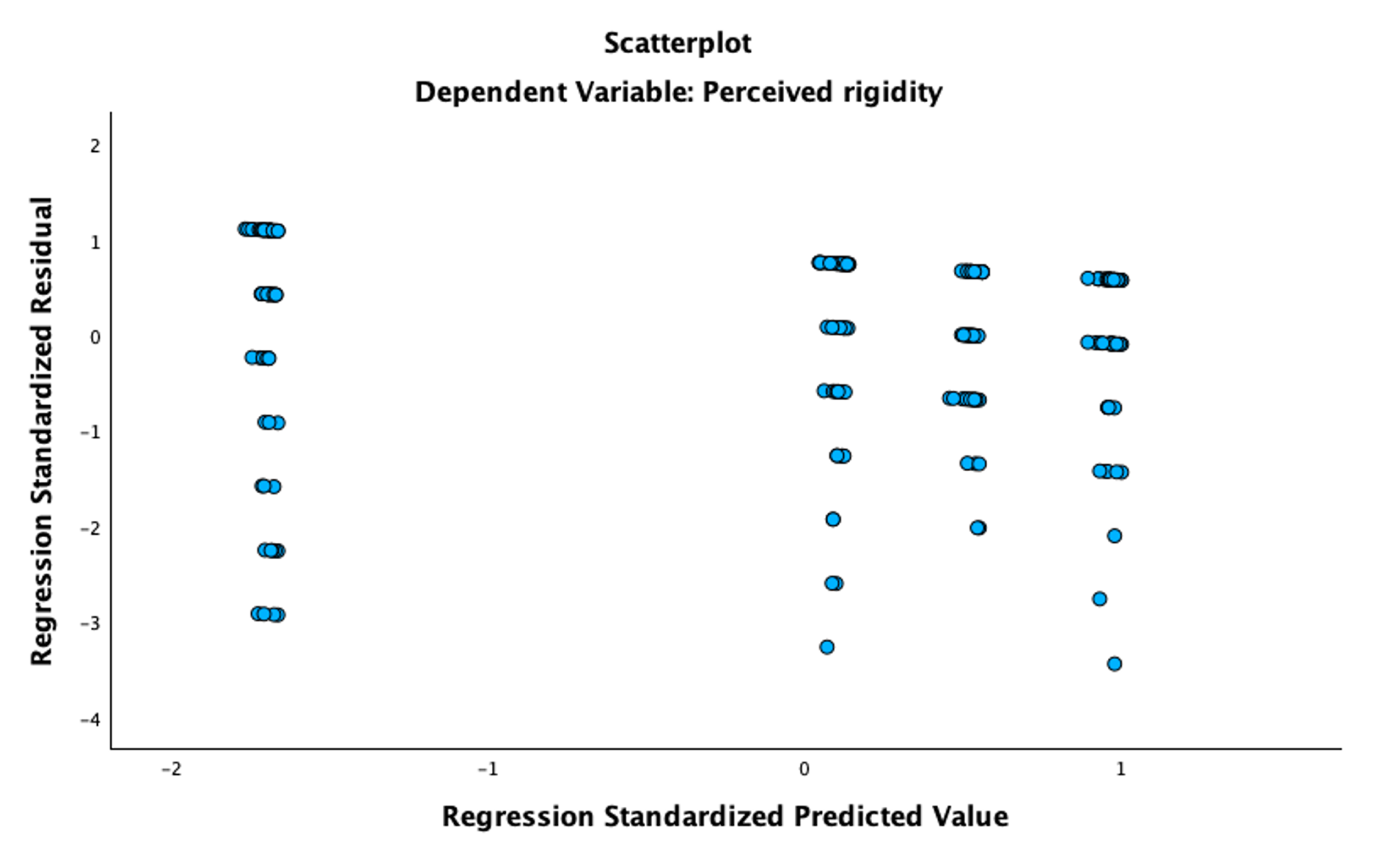


Because the assumption of homoscedasticity was not perfectly met, we reran our analysis with robust standard errors (HC3) to justify the interpretation of our analysis in PROCESS. The results of our model with HC3 are shown in Tables 1 and 2. Although the standard errors are higher compared to PROCESS, the same relationships were significant. We thus continue to interpret the model output from PROCESS.

**Table 1. Model predicting distributive justice in Study 2 with Parameter Estimates with Robust Standard Errors (HC3)**

|  | *B* | *Robust SE* | *p* | *CI* |
| --- | --- | --- | --- | --- |
| Constant | 2.44 | .80 | .00 | .86,4.03 |
| AI attitude | .23 | .06 | .00 | .11,.34 |
| Tenured position | .17 | .23 | .47 | -.29,.63 |
| Nonfulfillment | -.02 | .30 | .95 | -.61,.57 |
| Anthropomorphism | .35 | .27 | .20 | -.19,.89 |
| Nonfulfillment* anthropomorphism | -.21 | .40 | .61 | -.99,.58 |
| Rigidity | -.20 | .08 | .02 | -.36,-.04 |
| R^2^ | .03 |  | .24 |  |

**Table 2. Model predicting rigidity in Study 2 with Parameter Estimates with Robust Standard Errors (HC3)**

|  | *B* | *Robust SE* | *p* | *CI* |
| --- | --- | --- | --- | --- |
| Constant | 5.32 | .68 | .00 | 3.99,6.66 |
| AI attitude | .00 | .05 | .95 | -.10,.11 |
| Tenured position | .00 | .24 | .99 | -.47,.48 |
| Nonfulfillment | .77 | .31 | .01 | .16,1.83 |
| Anthropomorphism | .64 | .30 | .03 | .06,1.23 |
| Nonfulfillment* anthropomorphism | -.89 | .39 | .02 | -1.65,-.13 |
| R^2^ | .03 |  | .24 |  |

**Influence Diagnostics – Study 2**

For the model predicting distributive justice, studentized residuals range between -2.10 and 2.53. For the model predicting rigidity, studentized residuals range between -3.47 and 1.15. For both models, studentized residuals do not indicate a particular influence of single cases.

For the model predicting distributive justice, the values of leverage range between .01 and .08. Based on the number of predictors and the sample size, a threshold of .06 indicates a particular influence of single cases. Three cases in our study show an influence above the defined threshold. For the model predicting rigidity, the values of leverage range between .01 and .05. Based on the number of predictors and the sample size, a threshold of .05 indicates a particular influence of single cases.

For the model predicting distributive justice, Cook’s Distance ranges between .00 and .057. Based on the sample size, a threshold of .016 indicates a particular influence of single cases. Eleven cases lie above this threshold. For the model predicting rigidity, Cook’s Distance ranges between .00 and .069. Based on the sample size, a threshold of .016 indicates a particular influence of single cases. Fifteen cases lie above this threshold.

Based on the sample size, a threshold of .13 indicates a particularly high/low DFBETA and a related influence of this case. For both models, PROCESS identifies one case with a DFBETA higher than .13 in the constant. All other DFBETAs lie below this threshold.

**Moderation – Study 2**

**Johnson-Neyman Plot**

The Johnson-Neyman plot (Fig 5) for the interaction between anthropomorphism and nonfulfillment illustrates that the interaction is significant for low values of nonfulfillment only (i.e., relational non-fulfillment). The plot was produced with the package interactions in R (Long, 2024).

**Fig 5. Johnson-Neyman Plot**


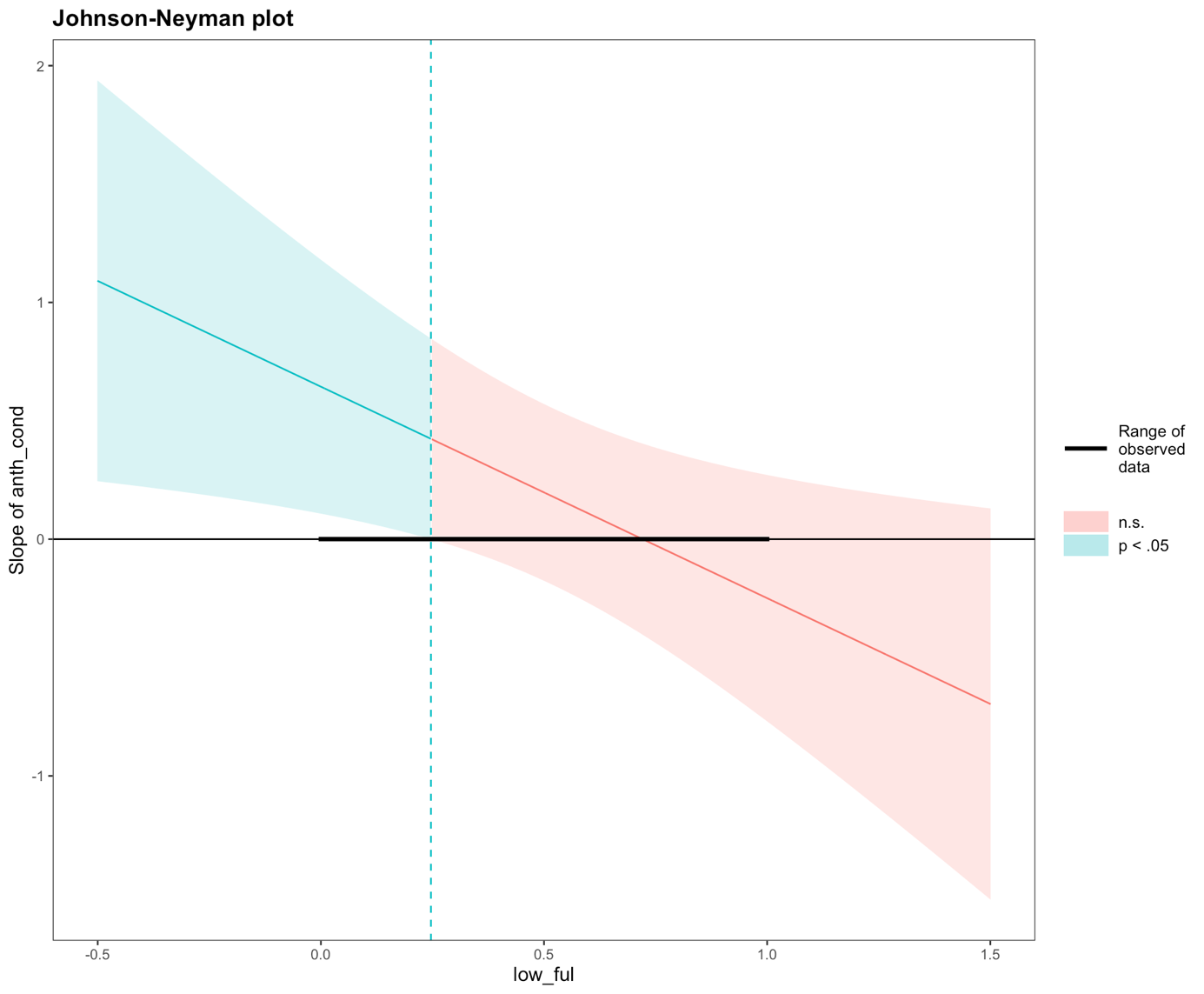


*Note.* Low_ful = nonfulfillment (0=relational, 1=transactional) ; anth_cond = anthropomorphism (0=low, 1=high)

**Reference**

Long J. A. (2024). interactions: comprehensive, user-friendly toolkit for probing interactions. R package version 1.2.0. [https://CRAN.R-project.org/package=interactions](https://cran.r-project.org/package=interactions)
